# The *FANCM*:p.Arg658* truncating variant is associated with risk of triple-negative breast cancer

**DOI:** 10.1038/s41523-019-0127-5

**Published:** 2019-11-01

**Authors:** Gisella Figlioli, Massimo Bogliolo, Irene Catucci, Laura Caleca, Sandra Viz Lasheras, Roser Pujol, Johanna I. Kiiski, Taru A. Muranen, Daniel R. Barnes, Joe Dennis, Kyriaki Michailidou, Manjeet K. Bolla, Goska Leslie, Cora M. Aalfs, ABCTB Investigators, Rosemary Balleine, Robert Baxter, Stephen Braye, Jane Carpenter, Jane Dahlstrom, John Forbes, C. Soon Lee, Deborah Marsh, Adrienne Morey, Nirmala Pathmanathan, Rodney Scott, Peter Simpson, Allan Spigelman, Nicholas Wilcken, Desmond Yip, Nikolajs Zeps, Muriel A. Adank, Julian Adlard, Simona Agata, Karen Cadoo, Bjarni A. Agnarsson, Thomas Ahearn, Kristiina Aittomäki, Christine B. Ambrosone, Lesley Andrews, Hoda Anton-Culver, Natalia N. Antonenkova, Volker Arndt, Norbert Arnold, Kristan J. Aronson, Banu K. Arun, Ella Asseryanis, Bernd Auber, Päivi Auvinen, Jacopo Azzollini, Judith Balmaña, Rosa B. Barkardottir, Daniel Barrowdale, Julian Barwell, Laura E. Beane Freeman, Charles Joly Beauparlant, Matthias W. Beckmann, Sabine Behrens, Javier Benitez, Raanan Berger, Marina Bermisheva, Amie M. Blanco, Carl Blomqvist, Natalia V. Bogdanova, Anders Bojesen, Stig E. Bojesen, Bernardo Bonanni, Ake Borg, Angela F. Brady, Hiltrud Brauch, Hermann Brenner, Thomas Brüning, Barbara Burwinkel, Saundra S. Buys, Trinidad Caldés, Almuth Caliebe, Maria A. Caligo, Daniele Campa, Ian G. Campbell, Federico Canzian, Jose E. Castelao, Jenny Chang-Claude, Stephen J. Chanock, Kathleen B. M. Claes, Christine L. Clarke, Anita Collavoli, Thomas A. Conner, David G. Cox, Cezary Cybulski, Kamila Czene, Mary B. Daly, Miguel de la Hoya, Peter Devilee, Orland Diez, Yuan Chun Ding, Gillian S. Dite, Nina Ditsch, Susan M. Domchek, Cecilia M. Dorfling, Isabel dos-Santos-Silva, Katarzyna Durda, Miriam Dwek, Diana M. Eccles, Arif B. Ekici, A. Heather Eliassen, Carolina Ellberg, Mikael Eriksson, D. Gareth Evans, Peter A. Fasching, Jonine Figueroa, Henrik Flyger, William D. Foulkes, Tara M. Friebel, Eitan Friedman, Marike Gabrielson, Pragna Gaddam, Manuela Gago-Dominguez, Chi Gao, Susan M. Gapstur, Judy Garber, Montserrat García-Closas, José A. García-Sáenz, Mia M. Gaudet, Simon A. Gayther, GEMO Study Collaborators, Muriel Belotti, Ophélie Bertrand, Anne-Marie Birot, Bruno Buecher, Sandrine Caputo, Anaïs Dupré, Emmanuelle Fourme, Marion Gauthier-Villars, Lisa Golmard, Marine Le Mentec, Virginie Moncoutier, Antoine de Pauw, Claire Saule, Nadia Boutry-Kryza, Alain Calender, Sophie Giraud, Mélanie Léone, Brigitte Bressac-de-Paillerets, Olivier Caron, Marine Guillaud-Bataille, Yves-Jean Bignon, Nancy Uhrhammer, Valérie Bonadona, Christine Lasset, Pascaline Berthet, Laurent Castera, Dominique Vaur, Violaine Bourdon, Catherine Noguès, Tetsuro Noguchi, Cornel Popovici, Audrey Remenieras, Hagay Sobol, Isabelle Coupier, Pascal Pujol, Claude Adenis, Aurélie Dumont, Françoise Révillion, Danièle Muller, Emmanuelle Barouk-Simonet, Françoise Bonnet, Virginie Bubien, Michel Longy, Nicolas Sevenet, Laurence Gladieff, Rosine Guimbaud, Viviane Feillel, Christine Toulas, Hélène Dreyfus, Christine Dominique Leroux, Magalie Peysselon, Christine Rebischung, Clémentine Legrand, Amandine Baurand, Geoffrey Bertolone, Fanny Coron, Laurence Faivre, Caroline Jacquot, Sarab Lizard, Caroline Kientz, Marine Lebrun, Fabienne Prieur, Sandra Fert-Ferrer, Véronique Mari, Laurence Vénat-Bouvet, Stéphane Bézieau, Capucine Delnatte, Isabelle Mortemousque, Chrystelle Colas, Florence Coulet, Florent Soubrier, Mathilde Warcoin, Myriam Bronner, Johanna Sokolowska, Marie-Agnès Collonge-Rame, Alexandre Damette, Paul Gesta, Hakima Lallaoui, Jean Chiesa, Denise Molina-Gomes, Olivier Ingster, Sylvie Manouvrier-Hanu, Sophie Lejeune, Graham G. Giles, Gord Glendon, Andrew K. Godwin, Mark S. Goldberg, David E. Goldgar, Pascal Guénel, Angelica M. Gutierrez-Barrera, Lothar Haeberle, Christopher A. Haiman, Niclas Håkansson, Per Hall, Ute Hamann, Patricia A. Harrington, Alexander Hein, Jane Heyworth, Peter Hillemanns, Antoinette Hollestelle, John L. Hopper, H. Dean Hosgood, Anthony Howell, Chunling Hu, Peter J. Hulick, David J. Hunter, Evgeny N. Imyanitov, KConFab, Morteza Aghmesheh, Sian Greening, David Amor, Mike Gattas, Leon Botes, Michael Buckley, Michael Friedlander, Jessica Koehler, Bettina Meiser, Mona Saleh, Elizabeth Salisbury, Alison Trainer, Kathy Tucker, Yoland Antill, Alexander Dobrovic, Andrew Fellows, Stephen Fox, Marion Harris, Sophie Nightingale, Kelly Phillips, Joe Sambrook, Heather Thorne, Shane Armitage, Leanne Arnold, Rosemary Balleine, Rick Kefford, Judy Kirk, Edwina Rickard, Patti Bastick, Jonathan Beesley, Nick Hayward, Amanda Spurdle, Logan Walker, John Beilby, Christobel Saunders, Ian Bennett, Anneke Blackburn, Michael Bogwitz, Clara Gaff, Geoff Lindeman, Nick Pachter, Clare Scott, Adrienne Sexton, Jane Visvader, Jessica Taylor, Ingrid Winship, Meagan Brennan, Melissa Brown, Juliet French, Stacey Edwards, Matthew Burgess, Jo Burke, Briony Patterson, Phyllis Butow, Bronwyn Culling, Liz Caldon, David Callen, Deepa Chauhan, Maurice Eisenbruch, Louise Heiniger, Manisha Chauhan, Alice Christian, Joanne Dixon, Alexa Kidd, Paul Cohen, Alison Colley, Georgina Fenton, Ashley Crook, Rebecca Dickson, Michael Field, Deborah Marsh, James Cui, Margaret Cummings, Sarah-Jane Dawson, Anna DeFazio, Martin Delatycki, Tracy Dudding, Ted Edkins, Gelareh Farshid, James Flanagan, Peter Fong, Laura Forrest, David Gallego-Ortega, Peter George, Grantley Gill, James Kollias, Eric Haan, Stewart Hart, Mark Jenkins, Clare Hunt, Sunil Lakhani, Lara Lipton, Liz Lobb, Graham Mann, Sue Anne McLachlan, Shona O’Connell, Sarah O’Sullivan, Ellen Pieper, Bridget Robinson, Jodi Saunus, Elizabeth Scott, Rodney Scott, Andrew Shelling, Peter Simpson, Rachael Williams, Mary Ann Young, Claudine Isaacs, Milena Jakimovska, Anna Jakubowska, Paul James, Ramunas Janavicius, Wolfgang Janni, Esther M. John, Michael E. Jones, Audrey Jung, Rudolf Kaaks, Beth Y. Karlan, Elza Khusnutdinova, Cari M. Kitahara, Irene Konstantopoulou, Stella Koutros, Peter Kraft, Diether Lambrechts, Conxi Lazaro, Loic Le Marchand, Jenny Lester, Fabienne Lesueur, Jenna Lilyquist, Jennifer T. Loud, Karen H. Lu, Robert N. Luben, Jan Lubinski, Arto Mannermaa, Mehdi Manoochehri, Siranoush Manoukian, Sara Margolin, John W. M. Martens, Tabea Maurer, Dimitrios Mavroudis, Noura Mebirouk, Alfons Meindl, Usha Menon, Austin Miller, Marco Montagna, Katherine L. Nathanson, Susan L. Neuhausen, William G. Newman, Tu Nguyen-Dumont, Finn Cilius Nielsen, Sarah Nielsen, Liene Nikitina-Zake, Kenneth Offit, Edith Olah, Olufunmilayo I. Olopade, Andrew F. Olshan, Janet E. Olson, Håkan Olsson, Ana Osorio, Laura Ottini, Bernard Peissel, Ana Peixoto, Julian Peto, Dijana Plaseska-Karanfilska, Timea Pocza, Nadege Presneau, Miquel Angel Pujana, Kevin Punie, Brigitte Rack, Johanna Rantala, Muhammad U. Rashid, Rohini Rau-Murthy, Gad Rennert, Flavio Lejbkowicz, Valerie Rhenius, Atocha Romero, Matti A. Rookus, Eric A. Ross, Maria Rossing, Vilius Rudaitis, Matthias Ruebner, Emmanouil Saloustros, Kristin Sanden, Marta Santamariña, Maren T. Scheuner, Rita K. Schmutzler, Michael Schneider, Christopher Scott, Leigha Senter, Mitul Shah, Priyanka Sharma, Xiao-Ou Shu, Jacques Simard, Christian F. Singer, Christof Sohn, Penny Soucy, Melissa C. Southey, John J. Spinelli, Linda Steele, Dominique Stoppa-Lyonnet, William J. Tapper, Manuel R. Teixeira, Mary Beth Terry, Mads Thomassen, Jennifer Thompson, Darcy L. Thull, Marc Tischkowitz, Rob A.E.M. Tollenaar, Diana Torres, Melissa A. Troester, Thérèse Truong, Nadine Tung, Michael Untch, Celine M. Vachon, Elizabeth J. van Rensburg, Elke M. van Veen, Ana Vega, Alessandra Viel, Barbara Wappenschmidt, Jeffrey N. Weitzel, Camilla Wendt, Greet Wieme, Alicja Wolk, Xiaohong R. Yang, Wei Zheng, Argyrios Ziogas, Kristin K. Zorn, Alison M. Dunning, Michael Lush, Qin Wang, Lesley McGuffog, Michael T. Parsons, Paul D. P. Pharoah, Florentia Fostira, Amanda E. Toland, Irene L. Andrulis, Susan J. Ramus, Anthony J. Swerdlow, Mark H. Greene, Wendy K. Chung, Roger L. Milne, Georgia Chenevix-Trench, Thilo Dörk, Marjanka K. Schmidt, Douglas F. Easton, Paolo Radice, Eric Hahnen, Antonis C. Antoniou, Fergus J. Couch, Heli Nevanlinna, Jordi Surrallés, Paolo Peterlongo

**Affiliations:** 1 IFOM - the FIRC Institute for Molecular Oncology, Genome Diagnostics Program, Milan, Italy; 2 Department of Genetics and Microbiology, Universitat Autònoma de Barcelona, Bellaterra, Barcelona Spain; 3 Center for Biomedical Network Research on Rare Diseases (CIBERER), Madrid, Spain; 4 Institute of Biomedical Research, Sant Pau Hospital, Barcelona, Spain; 5 Fondazione IRCCS Istituto Nazionale dei Tumori, Unit of Molecular Bases of Genetic Risk and Genetic Testing, Department of Research, Milan, Italy; 6 University of Helsinki, Department of Obstetrics and Gynecology, Helsinki University Hospital, Helsinki, Finland; 7 University of Cambridge, Centre for Cancer Genetic Epidemiology, Department of Public Health and Primary Care, Cambridge, UK; 8 The Cyprus Institute of Neurology & Genetics, Department of Electron Microscopy/Molecular Pathology and The Cyprus School of Molecular Medicine, Nicosia, Cyprus; 9 Amsterdam UMC, lokatie AMC, Department of Clinical Genetics, Amsterdam, The Netherlands; 10 The Netherlands Cancer Institute - Antoni van Leeuwenhoek hospital, Family Cancer Clinic, Amsterdam, The Netherlands; 11 Chapel Allerton Hospital, Yorkshire Regional Genetics Service, Leeds, UK; 12 Veneto Institute of Oncology IOV - IRCCS, Immunology and Molecular Oncology Unit, Padua, Italy; 13 Memorial Sloan-Kettering Cancer Center, Department of Medicine, New York, NY USA; 14 Landspitali University Hospital, Department of Pathology, Reykjavik, Iceland; 15 University of Iceland, School of Medicine, Reykjavik, Iceland; 16 National Cancer Institute, National Institutes of Health, Department of Health and Human Services, Division of Cancer Epidemiology and Genetics, Bethesda, MD USA; 17 University of Helsinki, Department of Clinical Genetics, Helsinki University Hospital, Helsinki, Finland; 18 Roswell Park Cancer Institute, Buffalo, NY USA; 19 Nelune Comprehensive Cancer Care Centre, The Bright Alliance Building, Randwick, NSW Australia; 20 University of California Irvine, Department of Epidemiology, Genetic Epidemiology Research Institute, Irvine, CA USA; 21 N.N. Alexandrov Research Institute of Oncology and Medical Radiology, Minsk, Belarus; 22 German Cancer Research Center (DKFZ), Division of Clinical Epidemiology and Aging Research, Heidelberg, Germany; 23 University Hospital of Schleswig-Holstein, Campus Kiel, Christian-Albrechts University Kiel, Department of Gynaecology and Obstetrics, and Institute of Clinical Molecular Biology, Kiel, Germany; 24 Queen’s University, Department of Public Health Sciences, and Cancer Research Institute, Kingston, ON Canada; 25 University of Texas MD Anderson Cancer Center, Department of Breast Medical Oncology, Houston, TX USA; 26 Medical University of Vienna, Dept of OB/GYN and Comprehensive Cancer Center, Vienna, Austria; 27 Hannover Medical School, Institute of Human Genetics, Hannover, Germany; 28 Kuopio University Hospital, Cancer Center, Kuopio, Finland; 29 University of Eastern Finland, Institute of Clinical Medicine, Oncology, Kuopio, Finland; 30 University of Eastern Finland, Translational Cancer Research Area, Kuopio, Finland; 31 Fondazione IRCCS Istituto Nazionale dei Tumori di Milano, Department of Medical Oncology and Hematology, Unit of Medical Genetics, Milan, Italy; 32 Vall d’Hebron Institute of Oncology, High Risk and Cancer Prevention Group, Barcelona, Spain; 33 University Hospital, Vall d’Hebron, Department of Medical Oncology, Barcelona, Spain; 34 University of Iceland, BMC (Biomedical Centre), Faculty of Medicine, Reykjavik, Iceland; 35 University Hospitals of Leicester NHS Trust, Leicestershire Clinical Genetics Service, Leicester, UK; 36 Centre Hospitalier Universitaire de Québec – Université Laval, Research Center, Genomics Center, Québec City, QC Canada; 37 University Hospital Erlangen, Friedrich-Alexander-University Erlangen-Nuremberg, Department of Gynecology and Obstetrics, Comprehensive Cancer Center ER-EMN, Erlangen, Germany; 38 German Cancer Research Center (DKFZ), Division of Cancer Epidemiology, Heidelberg, Germany; 39 Spanish National Cancer Research Centre (CNIO), Human Genetics Group, Human Cancer Genetics Programme, Madrid, Spain; 40 Spanish Network on Rare Diseases (CIBERER), Madrid, Spain; 41 Spanish National Cancer Research Centre (CNIO), Genotyping Unit (CEGEN), Human Cancer Genetics Programme, Madrid, Spain; 42 Chaim Sheba Medical Center, The Institute of Oncology, Ramat Gan, Israel; 43 Ufa Federal Research Center of the Russian Academy of Sciences, Institute of Biochemistry and Genetics, Ufa, Russia; 44 University of California San Francisco, Cancer Genetics and Prevention Program, San Francisco, CA USA; 45 University of Helsinki, Department of Oncology, Helsinki University Hospital, Helsinki, Finland; 46 Örebro University Hospital, Department of Oncology, Örebro, Sweden; 47 Hannover Medical School, Department of Radiation Oncology, Hannover, Germany; 48 Hannover Medical School, Gynaecology Research Unit, Hannover, Germany; 49 Aarhus University Hospital, Department of Clinical Genetics, Aarhus, Denmark; 50 Copenhagen University Hospital, Copenhagen General Population Study, Herlev and Gentofte Hospital, Herlev, Denmark; 51 Copenhagen University Hospital, Department of Clinical Biochemistry, Herlev and Gentofte Hospital, Herlev, Denmark; 52 University of Copenhagen, Faculty of Health and Medical Sciences, Copenhagen, Denmark; 53 IEO, European Institute of Oncology IRCCS, Division of Cancer Prevention and Genetics, Milan, Italy; 54 Lund University and Skåne University Hospital, Department of Oncology, Lund, Sweden; 55 London North West University Hospitals NHS Trust, Northwick Park Hospital, North West Thames Regional Genetics Service, Kennedy Galton Centre, Harrow, UK; 56 Dr. Margarete Fischer-Bosch-Institute of Clinical Pharmacology, Stuttgart, Germany; 57 University of Tübingen, iFIT-Cluster of Excellence, Tübingen, Germany; 58 German Cancer Research Center (DKFZ), German Cancer Consortium (DKTK), Heidelberg, Germany; 59 German Cancer Research Center (DKFZ) and National Center for Tumor Diseases (NCT), Division of Preventive Oncology, Heidelberg, Germany; 60 Institute for Prevention and Occupational Medicine of the German Social Accident Insurance, Institute of the Ruhr University Bochum, Bochum, Germany; 61 German Cancer Research Center (DKFZ), Molecular Epidemiology Group, C080 Heidelberg, Germany; 62 University of Heidelberg, Molecular Biology of Breast Cancer, University Womens Clinic Heidelberg, Heidelberg, Germany; 63 Huntsman Cancer Institute, Department of Medicine, Salt Lake City, UT USA; 64 Instituto de Investigación Sanitaria San Carlos (IdISSC), Centro Investigación Biomédica en Red de Cáncer (CIBERONC), Medical Oncology Department, Hospital Clínico San Carlos, Madrid, Spain; 65 University Hospital of Schleswig-Holstein, Campus Kiel, Christian-Albrechts University Kiel, Institute of Human Genetics, Kiel, Germany; 66 University Hospital of Pisa, Section of Molecular Genetics, Dept. of Laboratory Medicine, Pisa, Italy; 67 University of Pisa, Department of Biology, Pisa, Italy; 68 Peter MacCallum Cancer Center, Research Division, Melbourne, VIC Australia; 69 The University of Melbourne, Sir Peter MacCallum Department of Oncology, Melbourne, VIC Australia; 70 German Cancer Research Center (DKFZ), Genomic Epidemiology Group, Heidelberg, Germany; 71 Instituto de Investigacion Sanitaria Galicia Sur (IISGS), Xerencia de Xestion Integrada de Vigo-SERGAS, Oncology and Genetics Unit, Vigo, Spain; 72 University Medical Center Hamburg-Eppendorf, Cancer Epidemiology Group, University Cancer Center Hamburg (UCCH), Hamburg, Germany; 73 Ghent University, Centre for Medical Genetics, Gent, Belgium; 74 University of Sydney, Westmead Institute for Medical Research, Sydney, NSW Australia; 75 University and University Hospital of Pisa, Section of Genetic Oncology, Dept. of Laboratory Medicine, Pisa, Italy; 76 Huntsman Cancer Institute, Salt Lake City, UT USA; 77 Imperial College London, Department of Epidemiology and Biostatistics, School of Public Health, London, UK; 78 Cancer Research Center of Lyon, INSERM, U1052 Lyon, France; 79 Pomeranian Medical University, Department of Genetics and Pathology, Szczecin, Poland; 80 Karolinska Institutet, Department of Medical Epidemiology and Biostatistics, Stockholm, Sweden; 81 Fox Chase Cancer Center, Department of Clinical Genetics, Philadelphia, PA USA; 82 Leiden University Medical Center, Department of Pathology, Leiden, The Netherlands; 83 Leiden University Medical Center, Department of Human Genetics, Leiden, The Netherlands; 84 Vall d’Hebron Institute of Oncology (VHIO), Oncogenetics Group, Barcelona, Spain; 85 University Hospital Vall d’Hebron, Area of Clinical and Molecular Genetics, Barcelona, Spain; 86 Beckman Research Institute of City of Hope, Department of Population Sciences, Duarte, CA USA; 87 The University of Melbourne, Centre for Epidemiology and Biostatistics, Melbourne School of Population and Global Health, Melbourne, Victoria, Australia; 88 Ludwig Maximilian University of Munich, Department of Gynecology and Obstetrics, Munich, Germany; 89 Perelman School of Medicine at the University of Pennsylvania, Department of Medicine, Abramson Cancer Center, Philadelphia, PA USA; 90 University of Pretoria, Department of Genetics, Arcadia, South Africa; 91 London School of Hygiene and Tropical Medicine, Department of Non-Communicable Disease Epidemiology, London, UK; 92 University of Westminster, Department of Biomedical Sciences, Faculty of Science and Technology, London, UK; 93 University of Southampton, Cancer Sciences Academic Unit, Faculty of Medicine, Southampton, UK; 94 Friedrich-Alexander University Erlangen-Nuremberg, Comprehensive Cancer Center Erlangen-EMN, Institute of Human Genetics, University Hospital Erlangen, Erlangen, Germany; 95 Harvard Medical School, Channing Division of Network Medicine, Department of Medicine, Brigham and Women’s Hospital, Boston, MA USA; 96 Harvard T.H. Chan School of Public Health, Department of Epidemiology, Boston, MA USA; 97 Lund University, Department of Cancer Epidemiology, Clinical Sciences, Lund, Sweden; 98 University of Manchester, Division of Evolution and Genomic Medicine, School of Biological Sciences, Faculty of Biology, Medicine and Health, Manchester, UK; 99 Manchester University NHS Foundation Trust, Manchester Academic Health Science Centre, Manchester Centre for Genomic Medicine, Manchester, UK; 100 University of California at Los Angeles, David Geffen School of Medicine, Department of Medicine Division of Hematology and Oncology, Los Angeles, CA USA; 101 The University of Edinburgh Medical School, Usher Institute of Population Health Sciences and Informatics, Edinburgh, UK; 102 Cancer Research UK Edinburgh Centre, Edinburgh, UK; 103 Copenhagen University Hospital, Department of Breast Surgery, Herlev and Gentofte Hospital, Herlev, Denmark; 104 McGill University, Program in Cancer Genetics, Departments of Human Genetics and Oncology, Montréal, QC Canada; 105 Harvard T.H. Chan School of Public Health, Boston, MA USA; 106 Dana-Farber Cancer Institute, Boston, MA USA; 107 Chaim Sheba Medical Center, The Susanne Levy Gertner Oncogenetics Unit, Ramat Gan, Israel; 108 Tel Aviv University, Sackler Faculty of Medicine, Ramat Aviv, Israel; 109 Memorial Sloan-Kettering Cancer Center, Clinical Genetics Research Lab, Department of Cancer Biology and Genetics, New York, NY USA; 110 Instituto de Investigación Sanitaria de Santiago de Compostela (IDIS), Complejo Hospitalario Universitario de Santiago, SERGAS, Genomic Medicine Group, Galician Foundation of Genomic Medicine, Santiago de Compostela, Spain; 111 University of California San Diego, Moores Cancer Center, La Jolla, CA USA; 112 Harvard T.H. Chan School of Public Health, Program in Genetic Epidemiology and Statistical Genetics, Boston, MA USA; 113 American Cancer Society, Epidemiology Research Program, Atlanta, GA USA; 114 Dana-Farber Cancer Institute, Cancer Risk and Prevention Clinic, Boston, MA USA; 115 The Institute of Cancer Research, Division of Genetics and Epidemiology, London, UK; 116 Cedars-Sinai Medical Center, The Center for Bioinformatics and Functional Genomics at the Samuel Oschin Comprehensive Cancer Institute, Los Angeles, CA USA; 117 Cancer Council Victoria, Cancer Epidemiology Division, Melbourne, VIC Australia; 118 Monash University, Department of Epidemiology and Preventive Medicine, Melbourne, VIC Australia; 119 Lunenfeld-Tanenbaum Research Institute of Mount Sinai Hospital, Fred A. Litwin Center for Cancer Genetics, Toronto, ON Canada; 120 Kansas University Medical Center, Department of Pathology and Laboratory Medicine, Kansas City, KS USA; 121 McGill University, Department of Medicine, Montréal, QC Canada; 122 McGill University, Division of Clinical Epidemiology, Royal Victoria Hospital, Montréal, QC Canada; 123 Huntsman Cancer Institute, University of Utah School of Medicine, Department of Dermatology, Salt Lake City, UT USA; 124 INSERM, University Paris-Sud, University Paris-Saclay, Cancer & Environment Group, Center for Research in Epidemiology and Population Health (CESP), Villejuif, France; 125 University of Texas MD Anderson Cancer Center, Department of Breast Medical Oncology and Clinical Genetics Program, Houston, TX USA; 126 Friedrich-Alexander University Erlangen-Nuremberg, Comprehensive Cancer Center Erlangen-EMN, Department of Gynaecology and Obstetrics, University Hospital Erlangen, Erlangen, Germany; 127 University of Southern California, Department of Preventive Medicine, Keck School of Medicine, Los Angeles, CA USA; 128 Karolinska Institutet, Institute of Environmental Medicine, Stockholm, Sweden; 129 Södersjukhuset, Department of Oncology, Stockholm, Sweden; 130 German Cancer Research Center (DKFZ), Molecular Genetics of Breast Cancer, Heidelberg, Germany; 131 University of Cambridge, Centre for Cancer Genetic Epidemiology, Department of Oncology, Cambridge, UK; 132 The University of Western Australia, School of Population and Global Health, Perth, WA Australia; 133 Erasmus MC Cancer Institute, Department of Medical Oncology, Family Cancer Clinic, Rotterdam, The Netherlands; 134 Albert Einstein College of Medicine, Department of Epidemiology and Public Health, Bronx, NY USA; 135 University of Manchester, Division of Cancer Sciences, Manchester, UK; 136 Mayo Clinic, Department of Laboratory Medicine and Pathology, Rochester, MN USA; 137 NorthShore University HealthSystem, Center for Medical Genetics, Evanston, IL USA; 138 The University of Chicago Pritzker School of Medicine, Chicago, IL USA; 139 University of Oxford, Nuffield Department of Population Health, Oxford, UK; 140 N.N. Petrov Institute of Oncology, St. Petersburg, Russia; 141 Lombardi Comprehensive Cancer Center, Georgetown University, Washington, DC USA; 142 Macedonian Academy of Sciences and Arts, Research Centre for Genetic Engineering and Biotechnology ‘Georgi D. Efremov’, Skopje, Republic of Macedonia; 143 Pomeranian Medical University, Independent Laboratory of Molecular Biology and Genetic Diagnostics, Szczecin, Poland; 144 Peter MacCallum Cancer Center, Parkville Familial Cancer Centre, Melbourne, VIC Australia; 145 Vilnius University Hospital Santariskiu Clinics, Hematology, oncology and transfusion medicine center, Dept. of Molecular and Regenerative Medicine, Vilnius, Lithuania; 146 State Research Institute Innovative Medicine Center, Vilnius, Lithuania; 147 University Hospital Ulm, Department of Gynaecology and Obstetrics, Ulm, Germany; 148 Stanford University School of Medicine, Department of Medicine (Oncology) and Stanford Cancer Institute, Stanford, CA USA; 149 Cedars-Sinai Medical Center, Women’s Cancer Program at the Samuel Oschin Comprehensive Cancer Institute, Los Angeles, CA USA; 150 Bashkir State Medical University, Department of Medical Genetics, Ufa, Russia; 151 National Cancer Institute, Radiation Epidemiology Branch, Division of Cancer Epidemiology and Genetics, Bethesda, MD USA; 152 National Centre for Scientific Research ‘Demokritos’, Molecular Diagnostics Laboratory, INRASTES, Athens, Greece; 153 VIB, VIB Center for Cancer Biology, Leuven, Belgium; 154 University of Leuven, Laboratory for Translational Genetics, Department of Human Genetics, Leuven, Belgium; 155 IDIBELL (Bellvitge Biomedical Research Institute),Catalan Institute of Oncology, CIBERONC, Molecular Diagnostic Unit, Hereditary Cancer Program, Barcelona, Spain; 156 University of Hawaii Cancer Center, Epidemiology Program, Honolulu, HI USA; 157 Inserm U900, Genetic Epidemiology of Cancer team, Paris, France; 158 PSL University, Paris, France; 159 Institut Curie, Paris, France; 160 Mines ParisTech, Fontainebleau, France; 161 Mayo Clinic, Department of Health Sciences Research, Rochester, MN USA; 162 National Cancer Institute, Clinical Genetics Branch, Division of Cancer Epidemiology and Genetics, Bethesda, MD USA; 163 University of Texas MD Anderson Cancer Center, Department of Gynecologic Oncology and Clinical Cancer Genetics Program, Houston, TX USA; 164 University of Cambridge, Clinical Gerontology, Department of Public Health and Primary Care, Cambridge, UK; 165 University of Eastern Finland, Institute of Clinical Medicine, Pathology and Forensic Medicine, Kuopio, Finland; 166 Kuopio University Hospital, Imaging Center, Department of Clinical Pathology, Kuopio, Finland; 167 Karolinska Institutet, Department of Clinical Science and Education, Södersjukhuset, Stockholm, Sweden; 168 University Hospital of Heraklion, Department of Medical Oncology, Heraklion, Greece; 169 University College London, MRC Clinical Trials Unit at UCL, Institute of Clinical Trials & Methodology, London, UK; 170 Roswell Park Cancer Institute, NRG Oncology, Clinical Trials Development Division, Buffalo, NY USA; 171 Monash University, Precision Medicine, School of Clinical Sciences at Monash Health, Clayton, VIC Australia; 172 Rigshospitalet, Copenhagen University Hospital, Center for Genomic Medicine, Copenhagen, Denmark; 173 The University of Chicago, Center for Clinical Cancer Genetics, Chicago, IL USA; 174 Latvian Biomedical Research and Study Centre, Riga, Latvia; 175 Memorial Sloan-Kettering Cancer Center, Clinical Genetics Service, Department of Medicine, New York, NY USA; 176 National Institute of Oncology, Department of Molecular Genetics, Budapest, Hungary; 177 University of North Carolina at Chapel Hill, Department of Epidemiology, Lineberger Comprehensive Cancer Center, Chapel Hill, NC USA; 178 University La Sapienza, Department of Molecular Medicine, Rome, Italy; 179 Portuguese Oncology Institute, Department of Genetics, Porto, Portugal; 180 IDIBELL (Bellvitge Biomedical Research Institute),Catalan Institute of Oncology, CIBERONC, ProCURE, Oncobell, Barcelona, Spain; 181 Leuven Cancer Institute, University Hospitals Leuven, Multidisciplinary Breast Center, Department of General Medical Oncology, Leuven, Belgium; 182 Karolinska Institutet, Clinical Genetics, Stockholm, Sweden; 183 Shaukat Khanum Memorial Cancer Hospital and Research Centre (SKMCH & RC), Department of Basic Sciences, Lahore, Pakistan; 184 Carmel Medical Center and Technion Faculty of Medicine, Clalit National Cancer Control Center, Haifa, Israel; 185 Hospital Universitario Puerta de Hierro, Medical Oncology Department, Madrid, Spain; 186 The Netherlands Cancer Institute, Department of Epidemiology, Amsterdam, The Netherlands; 187 Fox Chase Cancer Center, Biostatistics and Bioinformatics Facility, Philadelphia, PA USA; 188 Vilnius University, Medical Faculty, Institute of Clinical Medicine, Vilnius, Lithuania; 189 University Hospital of Larissa, Department of Oncology, Larissa, Greece; 190 University of Wisconsin, Cancer Center at ProHealth Care, Waukesha, WI USA; 191 Fundación Pública Galega Medicina Xenómica, Santiago De Compostela, Spain; 192 Instituto de Investigación Sanitaria de Santiago de Compostela, Santiago De Compostela, Spain; 193 University Hospital of Cologne, Center for Hereditary Breast and Ovarian Cancer, Cologne, Germany; 194 University of Cologne, Center for Molecular Medicine Cologne (CMMC), Cologne, Germany; 195 The Ohio State University, Clinical Cancer Genetics Program, Division of Human Genetics, Department of Internal Medicine, The Comprehensive Cancer Center, Columbus, OH USA; 196 University of Kansas Medical Center, Department of Internal Medicine, Division of Oncology, Westwood, KS USA; 197 Vanderbilt University School of Medicine, Division of Epidemiology, Department of Medicine, Vanderbilt Epidemiology Center, Vanderbilt-Ingram Cancer Center, Nashville, TN USA; 198 University of Heidelberg, National Center for Tumor Diseases, Heidelberg, Germany; 199 The University of Melbourne, Department of Clinical Pathology, Melbourne, VIC Australia; 200 BC Cancer, Population Oncology, Vancouver, BC Canada; 201 University of British Columbia, School of Population and Public Health, Vancouver, BC Canada; 202 Institut Curie, Service de Génétique, Paris, France; 203 INSERM U830, Department of Tumour Biology, Paris, France; 204 Université Paris Descartes, Paris, France; 205 University of Southampton, Faculty of Medicine, Southampton, UK; 206 University of Porto, Biomedical Sciences Institute (ICBAS), Porto, Portugal; 207 Columbia University, Department of Epidemiology, Mailman School of Public Health, New York, NY USA; 208 Odense University Hospital, Department of Clinical Genetics, Odence C, Denmark; 209 Magee-Womens Hospital, University of Pittsburgh School of Medicine, Department of Medicine, Pittsburgh, PA USA; 210 University of Cambridge, Department of Medical Genetics, Cambridge, UK; 211 Leiden University Medical Center, Department of Surgery, Leiden, The Netherlands; 212 Pontificia Universidad Javeriana, Institute of Human Genetics, Bogota, Colombia; 213 Beth Israel Deaconess Medical Center, Department of Medical Oncology, Boston, MA USA; 214 Helios Clinics Berlin-Buch, Department of Gynecology and Obstetrics, Berlin, Germany; 215 Centro di Riferimento Oncologico di Aviano (CRO), IRCCS, Division of Functional onco-genomics and genetics, Aviano, Italy; 216 City of Hope, Clinical Cancer Genetics, Duarte, CA USA; 217 Uppsala University, Department of Surgical Sciences, Uppsala, Sweden; 218 Magee-Womens Hospital, University of Pittsburgh School of Medicine, Pittsburgh, PA USA; 219 QIMR Berghofer Medical Research Institute, Department of Genetics and Computational Biology, Brisbane, QLD Australia; 220 The Ohio State University, Department of Cancer Biology and Genetics, Columbus, OH USA; 221 University of Toronto, Department of Molecular Genetics, Toronto, ON Canada; 222 University of NSW Sydney, School of Women’s and Children’s Health, Faculty of Medicine, Sydney, NSW Australia; 223 The Kinghorn Cancer Centre, Garvan Institute of Medical Research, Sydney, NSW Australia; 224 The Institute of Cancer Research, Division of Genetics and Epidemiology, London, UK; 225 The Institute of Cancer Research, Division of Breast Cancer Research, London, UK; 226 Columbia University, Departments of Pediatrics and Medicine, New York, NY USA; 227 The Netherlands Cancer Institute - Antoni van Leeuwenhoek Hospital, Division of Molecular Pathology, Amsterdam, The Netherlands; 228 The Netherlands Cancer Institute - Antoni van Leeuwenhoek hospital, Division of Psychosocial Research and Epidemiology, Amsterdam, The Netherlands; 229 Department of Genetics, Sant Pau Hospital, Barcelona, Spain; 230 Pathology West ICPMR, Westmead, NSW Australia; 231 Kolling Institute of Medical Research, University of Sydney, Royal North Shore Hospital, Sydney, NSW Australia; 232 Pathology North, John Hunter Hospital, Newcastle, NSW 2305 Australia; 233 Department of Anatomical Pathology, ACT Pathology, Canberra Hospital, Canberra, ACT Australia; 234 ANU Medical School, Australian National University, Canberra, ACT Australia; 235 Department of Surgical Oncology, Calvary Mater Newcastle Hospital, Australian New Zealand Breast Cancer Trials Group, and School of Medicine and Public Health, University of Newcastle, Newcastle, NSW Australia; 236 School of Science and Health, The University of Western Sydney, Sydney, NSW Australia; 237 Hormones and Cancer Group, Kolling Institute of Medical Research, Royal North Shore Hospital, University of Sydney, Sydney, NSW Australia; 238 SydPath St Vincent’s Hospital, Sydney, NSW Australia; 239 Department of Tissue Pathology and Diagnostic Oncology, Pathology West, Westmead Breast Cancer Institute, Westmead Hospital, Sydney, NSW Australia; 240 Centre for Information Based Medicine, Hunter Medical Research Institute, Sydney, NSW 2305 Australia; 241 Priority Research Centre for Cancer, School of Biomedical Sciences and Pharmacy, Faculty of Health, University of Newcastle, Callaghan, NSW Australia; 242 The University of Queensland, UQ Centre for Clinical Research and School of Medicine, Brisbane, QLD Australia; 243 Hereditary Cancer Clinic, St Vincent’s Hospital, The Kinghorn Cancer Centre, Sydney, NSW 2010 Australia; 244 Crown Princess Mary Cancer Centre, Westmead Hospital, Westmead, NSW Australia; 245 Sydney Medical School - Westmead, University of Sydney, Sydney, NSW Australia; 246 Department of Medical Oncology, The Canberra Hospital, Garran, ACT Australia; 247 St John of God Perth Northern Hospitals, Perth, WA Australia; 248 Unité Mixte de Génétique Constitutionnelle des Cancers Fréquents, Hospices Civils de Lyon, Lyon, France; 249 Centre Léon Bérard, Lyon, France; 250 Institut Gustave Roussy, Villejuif, France; 251 Centre Jean Perrin, Clermont–Ferrand, France; 252 Centre François Baclesse, Caen, France; 253 Institut Paoli Calmettes, Marseille, France; 254 CHU Arnaud-de-Villeneuve, Montpellier, France; 255 Centre Oscar Lambret, Lille, France; 256 Centre Paul Strauss, Strasbourg, France; 257 Institut Bergonié, Bordeaux, France; 258 Institut Claudius Regaud, Toulouse, France; 259 CHU, Grenoble, France; 260 CHU, Dijon, France; 261 CHU, St-Etienne, France; 262 Hôtel Dieu Centre Hospitalier, Chambéry, France; 263 Centre Antoine Lacassagne, Nice, France; 264 CHU, Limoges, France; 265 CHU, Nantes, France; 266 CHU Bretonneau, Tours and Centre Hospitalier de Bourges, Bourges, France; 267 Groupe Hospitalier Pitié-Salpétrière, Paris, France; 268 CHU Vandoeuvre-les-, Nancy, France; 269 CHU, Besançon, France; 270 CHU Poitiers, Centre Hospitalier d’Angoulême and Centre Hospitalier de Niort, Niort, France; 271 Centre Hospitalier de La Rochelle, La Rochelle, France; 272 CHU Nîmes, Carémeau, France; 273 CHI, Poissy, France; 274 CHU, Angers, France; 275 CHRU, de Lille, France; 276 Illawarra Cancer Care Centre Wollongong Hospital, Wollongong, Australia; 277 Royal Children’s Hospital, Melbourne, VIC Australia; 278 Prince of Wales Hospital, Randwick, NSW Australia; 279 Peter MacCallum Cancer Centre, East Melbourne, VIC Australia; 280 Royal Brisbane and Women’s Hospital, Herston, QLD 4029 Australia; 281 Westmead Hospital, Westmead, NSW Australia; 282 St George Hospital, Kogarah, NSW Australia; 283 Queensland Institute of Medical Research, Herston, QLD Australia; 284 Queen Elizabeth Medical Centre, Nedlands, WA Australia; 285 Silverton Place, Brisbane, QLD Australia; 286 Australian National University, Canberra, Australia; 287 The Royal Melbourne Hospital, Parkville, VIC Australia; 288 NSW Breast Cancer Institute, Westmead, NSW Australia; 289 University of Queensland, Queensland, QLD Australia; 290 Austin Health, Heidelberg, VIC Australia; 291 Royal Hobart Hospital, Hobart, TAS Australia; 292 Royal Prince Alfred Hospital, Camperdown, NSW Australia; 293 Garvan Institute of Medical Research, Darlinghurst, NSW Australia; 294 University of Adelaide/Hanson Institute, Rundle Mall, SA Australia; 295 University of Sydney, Sydney, NSW Australia; 296 The Kinghorn Cancer Centre, Sydney, NSW Australia; 297 Wellington Hospital, Wellington, New Zealand; 298 St John of God Subiaco Hospital, Subiaco, New Zealand; 299 Liverpool Health Service, Liverpool, UK; 300 Royal North Shore Hospital, St Leonards, NSW Australia; 301 Monash University, Melbourne, VIC Australia; 302 University of Queensland Medical School, Herston, NSW Australia; 303 Cambridge University, Cambridge, UK; 304 Westmead Institute for Cancer Research, Westmead, NSW Australia; 305 Heidelberg Repatriation Hospital, Heidelberg Heights, VIC Australia; 306 Hunter Area Health Service, Waratah, USA; 307 Princess Margret Hospital for Children, Perth, WA Australia; 308 IMVS, Adelaide, SA Australia; 309 Imperial College London, London, UK; 310 Auckland City Hospital, Auckland, New Zealand; 311 Parkville Familial Cancer Centre, Melbourne, VIC Australia; 312 The Kinghorn Cancer Centre, Darlinghurst, NSW Australia; 313 Canterbury Health Labs, Christchurch, New Zealand; 314 Royal Adelaide Hospital, Adelaide, SA Australia; 315 Women’s and Children’s Hospital, North Adelaide, NSW Australia; 316 Monash Medical Centre, Bentleigh, VIC Australia; 317 University of Melbourne, Melbourne, VIC Australia; 318 Monash Medical Centre, Melbourne, VIC Australia; 319 The Royal Brisbane & Women’s Hospital, Herston, QLD Australia; 320 Western Hospital, Footscray, VIC Australia; 321 Westmead Millennium Institute, Westmead, NSW Australia; 322 St Vincent’s Hospital, Fitzroy, VIC Australia; 323 Southern Health Familial Cancer Centre, Clayton, VIC USA; 324 Agnes Walsh House, Subiaco, WA Australia; 325 Genomic Medicine, Melbourne, VIC Australia; 326 Christchurch Hospital, Christchurch, New Zealand; 327 Women’s Hospital, Herston, QLD Australia; 328 South View Clinic, Kogarah, NSW Australia; 329 John Hunter Hospital, New Lambton Heights, NSW 2305 Australia; 330 University of Auckland, Auckland, New Zealand; 331 St Vincent’s Hospital, Darlinghurst, NSW Australia; 332 Genome.One, Darlinghurst, NSW Australia

**Keywords:** Cancer genetics, Cancer genetics, Cancer genetics

## Abstract

Breast cancer is a common disease partially caused by genetic risk factors. Germline pathogenic variants in DNA repair genes *BRCA1*, *BRCA2*, *PALB2*, *ATM*, and *CHEK2* are associated with breast cancer risk. *FANCM*, which encodes for a DNA translocase, has been proposed as a breast cancer predisposition gene, with greater effects for the ER-negative and triple-negative breast cancer (TNBC) subtypes. We tested the three recurrent protein-truncating variants *FANCM*:p.Arg658*, p.Gln1701*, and p.Arg1931* for association with breast cancer risk in 67,112 cases, 53,766 controls, and 26,662 carriers of pathogenic variants of *BRCA1* or *BRCA2*. These three variants were also studied functionally by measuring survival and chromosome fragility in *FANCM*^*−/−*^ patient-derived immortalized fibroblasts treated with diepoxybutane or olaparib. We observed that *FANCM*:p.Arg658* was associated with increased risk of ER-negative disease and TNBC (OR = 2.44, *P* = 0.034 and OR = 3.79; *P* = 0.009, respectively). In a country-restricted analysis, we confirmed the associations detected for *FANCM*:p.Arg658* and found that also *FANCM*:p.Arg1931* was associated with ER-negative breast cancer risk (OR = 1.96; *P* = 0.006). The functional results indicated that all three variants were deleterious affecting cell survival and chromosome stability with *FANCM*:p.Arg658* causing more severe phenotypes. In conclusion, we confirmed that the two rare *FANCM* deleterious variants p.Arg658* and p.Arg1931* are risk factors for ER-negative and TNBC subtypes. Overall our data suggest that the effect of truncating variants on breast cancer risk may depend on their position in the gene. Cell sensitivity to olaparib exposure, identifies a possible therapeutic option to treat *FANCM*-associated tumors.

## Introduction

The genetic architecture of inherited breast cancer is complex and involves germline pathogenic variants in high and moderate-risk genes and polygenetic factors. The major high-penetrance breast cancer risk genes include *BRCA1* and *BRCA2*, which are key factors in the DNA double-strand break repair through homologous recombination (HR) and in the inter-strand crosslink (ICL) repair as a part of the Fanconi Anemia (FA) pathway.^1,2^ Recently, based on a prospective cohort of families carrying *BRCA1* or *BRCA2* pathogenic variants, the average cumulative risk by age 80 was estimated to be 72% and 69% for carriers of *BRCA1* and *BRCA2* pathogenic variants, respectively.^3^ *PALB2* has been previously considered a moderate-risk gene, but the latest estimate of about 44% lifetime risk associated with pathogenic variants may raise this gene to the high-risk group.^4^ Pathogenic variants in moderate-penetrance genes *ATM* and *CHEK2* are also associated with breast cancer, conferring a 20% average lifetime risk.^5,6^ Recently, *BARD1*, *RAD51D, BRIP1*, and *RAD51C* have been proposed as risk factors for triple-negative breast cancer (TNBC) with *BARD1* and *RAD51D* conferring high risk, and *BRIP1* and *RAD51C* associated with moderate risk.^7^ Thus, the risk associated with pathogenic variants in each gene may vary by breast tumor subtype.

Many of the *BRCA/FA* pathway genes when altered by biallelic mutations cause FA disease. The *FANCM* gene (FA complementation group M, OMIM #609644) encodes for a translocase, which is a member of the BRCA/FA molecular pathway but has been recently disqualified as a disease-causing factor for FA.^8,9^ Some protein-truncating variants in the *FANCM* gene were described as moderate breast cancer risk factors with a greater risk of TNBC. In the Finnish population, *FANCM*:c.5101 C > T (p.Gln1701*, rs147021911) is relatively frequent and was reported to be associated with breast cancer with odds ratio (OR) of 1.86 with 95% confidence intervals (CIs) = 1.26–2.75. A larger effect was observed in familial cases (OR = 2.11; 95% CI = 1.43–3.32), for estrogen receptor-negative (ER-negative) breast cancer (OR = 2.37; 95% CI = 1.37–4.12) and for TNBC (OR = 3.56; 95% CI = 1.81–6.98).^10^ We showed an increased risk (OR = 3.93; 95% CI = 1.28–12.11) of the *FANCM*:c.5791 C > T (rs144567652) truncating variant using familial cases and controls. In vitro analysis showed that this variant causes the skipping of the *FANCM* exon 22 and the creation of a downstream stop codon (p.Gly1906Alafs12*).^11^ However, in the present study we refer to the *FANCM*:c.5791 C > T base change as to *FANCM*:p.Arg1931*, which is the conventional amino acid annotation (consistent with the stop codon creation according to genetic code). The *FANCM*:p.Arg1931* was also found to be associated with TNBC risk in the Finnish population (OR = 5.14; 95% CI = 1.65–16.0).^12^ A burden analysis of truncating variants discovered by a re-sequencing analysis of the entire *FANCM* coding region in German cases and controls confirmed that *FANCM* pathogenic variants had a particularly high risk for TNBC (OR = 3.75; 95% CI = 1.0–12.85).^13^

To study the effect of *FANCM* on breast cancer risk further, we tested three recurrent truncating variants *FANCM*:p.Arg658*, p.Gln1701*, and p.Arg1931*, within the OncoArray Consortium, a collaboration of consortia established to discover germline genetic variants predisposing to different human cancers (e.g., breast, colon, lung, ovary, endometrium and prostate cancers).^14^ These three variants were tested for association with breast cancer risk in 67,112 breast cancer cases, 53,766 controls, and 26,662 carriers of pathogenic variants in *BRCA1* or *BRCA2*. We also studied the functional effect of these three variants after their lentiviral transduction into a *FANCM*^*−/−*^ patient-derived cell line in which we measured survival and chromosome fragility after exposure to diepoxybutane (DEB) or the poly (ADP-ribose) polymerase inhibitor (PARPi) olaparib.

## Results

### Case-control analyses

We analyzed the association of three *FANCM* truncating variants, p.Arg658*, p.Gln1701*, and p.Arg1931*, with breast cancer risk for each variant separately and using a burden analysis. We tested 67,112 invasive breast cancer cases and 53,766 controls collected by the Breast Cancer Association Consortium (BCAC, http://bcac.ccge.medschl.cam.ac.uk/) and 26,662 carriers of *BRCA1* or *BRCA2* pathogenic variants collected by the Consortium of Investigators of Modifiers of *BRCA1/2* (CIMBA, http://cimba.ccge.medschl.cam.ac.uk/), of whom 13,497 were affected with breast cancer and 13,165 were unaffected.

In the BCAC dataset we assessed the breast cancer risk associated with the *FANCM* variants in a primary overall analysis and in a restricted analysis including only countries in which the variant carrier frequencies were higher than the median of the frequencies. In these analyses we tested association with the variants in all available invasive breast cancer cases or in the ER-positive, ER-negative and TNBC subgroups (Table 1). In the overall analysis, no evidence of association was observed, either with the presence of any *FANCM* variant or with any of the three variants individually. However, *FANCM*:p.Arg658* showed a higher heterozygote frequency in ER-negative breast cancer cases (0.093%) than in controls (0.035%) with a greater than two-fold increased breast cancer risk (OR = 2.44, 95% CI = 1.12–5.34, *P* = 0.034). When only TNBC cases were considered, the association was stronger (OR = 3.79, 95% CI = 1.56–9.18, *P* = 0.009). No association with ER-negative breast cancer or TNBC was seen for p.Gln1701* or p.Arg1931* or for all mutations combined (Table 1). In the country-restricted analyses, we confirmed the association found for p.Arg658* with risk of ER-negative disease and TNBC (OR = 2.31, 95% CI = 1.05–5.07, *P* = 0.047 and OR = 3.56, 95% CI = 1.46–8.69, *P* = 0.011, respectively). The restricted set also provided evidence for an association between p.Arg1931* and ER-negative subgroup (OR = 1.96, 95% CI = 1.24–3.10, *P* = 0.006), though not for TNBC. No significant association was observed for p.Gln1701* with either subgroups (Table 1).

**Table 1:** Single-variant and burden analyses of *FANCM*:p.Arg658*, p.Gln1701* and p.Arg1931* truncating variants in overall and country-restricted invasive breast cancer cases and controls

	Overall					
Subgroup	Carriers	Non-carriers	Freq %	OR	95% CI	*P*
*FANCM*:p.Arg658*						
Controls	19	53,717	0.035	NA		
All cases	31	67,038	0.046	1.26	0.71–2.25	0.430
ER-positive	19	44,516	0.043	1.15	0.61–2.20	0.670
ER-negative	10	10,750	0.093	**2.44**	**1.12**–**5.34**	**0.034**
TNBC	7	4794	0.146	**3.79**	**1.56**–**9.18**	**0.009**
*FANCM*:p.Gln1701*						
Controls	122	53,635	0.229	NA		
All cases	155	66,951	0.232	1.09	0.85–1.38	0.798
ER-positive	97	44,467	0.218	1.02	0.78–1.34	0.893
ER-negative	21	10,748	0.204	0.97	0.61–1.56	0.369
TNBC	10	4794	0.229	1.09	0.57–2.10	0.149
*FANCM*:p.Arg1931*						
Controls	96	53,633	0.179	NA		
All cases	116	66,968	0.173	1.05	0.80–1.38	0.731
ER-positive	74	44,467	0.166	1.02	0.75–1.38	0.920
ER-negative	27	10,742	0.251	1.52	0.98–2.35	0.070
TNBC	10	4795	0.208	1.29	0.67–2.50	0.461
All variants^a^						
Controls	237	53,455	0.443	NA		
All cases	302	66,736	0.452	1.02	0.86–1.21	0.823
ER-positive	190	44,323	0.427	0.96	0.79–1.16	0.698
ER-negative	58	10,700	0.548	1.23	0.92–1.64	0.154
TNBC	27	4773	0.583	1.32	0.89–1.95	0.167

	Country-restricted					
Subgroup	Carriers	Non-carriers	Freq %	OR	95% CI	*P*
*FANCM*:p.Arg658*						
Controls	19	48,887	0.039	NA		
All cases	31	59,540	0.052	1.23	0.69–2.20	0.478
ER-positive	19	39,453	0.048	1.12	0.59–2.15	0.722
ER-negative	10	9613	0.104	**2.31**	**1.05**–**5.07**	**0.047**
TNBC	7	4283	0.163	**3.56**	**1.46**–**8.69**	**0.011**
*FANCM*:p.Gln1701*						
Controls	120	48,506	0.249	NA		
All cases	152	58,919	0.259	1.08	0.85–1.38	0.813
ER-positive	96	38,892	0.246	1.02	0.77–1.34	0.895
ER-negative	21	9558	0.230	0.97	0.60–1.56	0.368
TNBC	10	4197	0.261	1.09	0.56–2.10	0.150
*FANCM*:p.Arg1931*						
Controls	77	34,988	0.220	NA		
All cases	93	37,903	0.245	1.14	0.84–1.54	0.396
ER-positive	59	25,274	0.233	1.09	0.77–1.53	0.632
ER-negative	25	5920	0.421	**1.96**	**1.24**–**3.10**	**0.006**
TNBC	10	2614	0.381	1.77	0.91–3.45	0.116
All variants^b^						
Controls	NA					
All cases	NA					
ER-positive	NA					
ER-negative	NA					
TNBC	NA					

In bold are indicated the statistically significant results

*Freq* frequency, *OR* odds ratio *CI* confidence interval, *P* *P*-value, *TNBC* triple-negative breast cancer, *NA* not applicable

^a^The burden analyses were performed by univariate logistic regression

^b^These analyses were not possible in the country-restricted cases and controls as different countries were included for each variant. *P*-values were from Pearson chi-squared test

### Analyses of carriers of *BRCA1* or *BRCA2* pathogenic variants

We found no evidence of associations for *FANCM*:p.Arg658*, p.Gln1701*, and p.Arg1931* truncating variants with breast cancer risk in carriers of *BRCA1* or *BRCA2* pathogenic variants included in CIMBA (Supplementary Table 1). The p.Arg658* was detected with approximately four-fold higher frequencies in the *BRCA1* affected individuals (0.063%) in comparison to the unaffected (0.013%), and in the *BRCA2* affected individuals (0.071%) in comparison to the unaffected (0.019%). Consistently, hazard ratios (HRs) above two were estimated for *BRCA1* (HR = 2.4, 95% CI = 0.52–11.12) and for *BRCA2* (HR = 2.13, 95% CI = 0.41–11.14) pathogenic variant carriers. The frequencies of p.Gln1701* and p.Arg1931* were not increased in affected versus unaffected individuals carrying *BRCA1* or *BRCA2* pathogenic variants (Supplementary Table 1).

### Functional studies

**Fig. 1:**
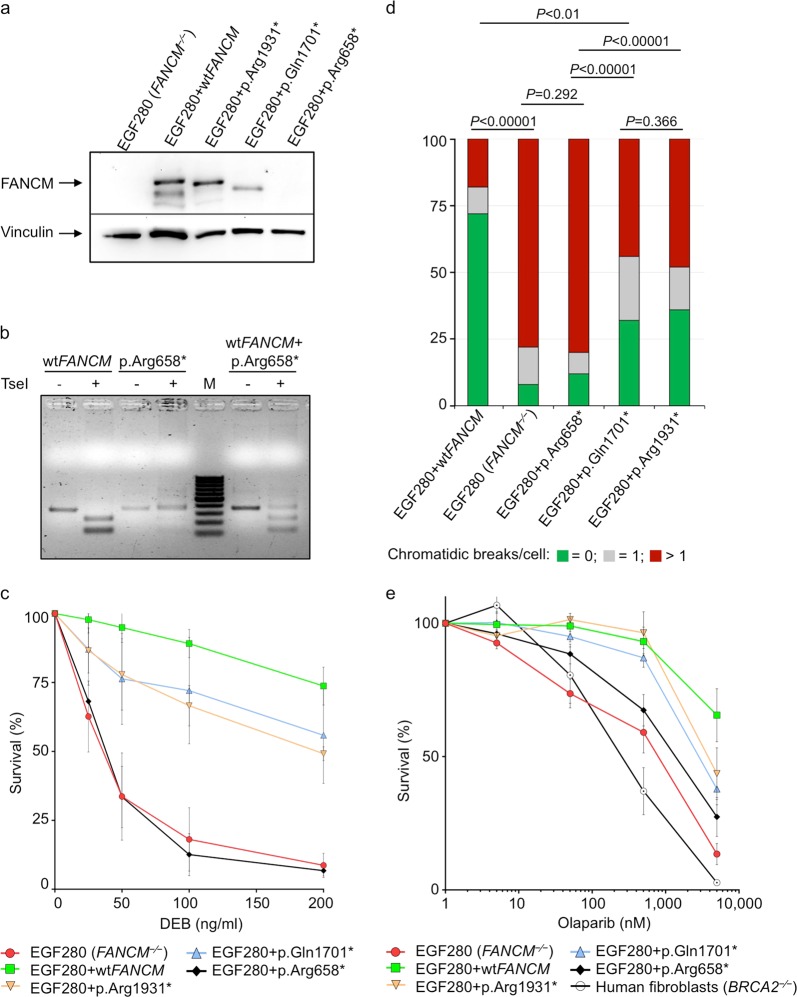
Functional studies of the *FANCM*:p.Arg658*, p.Gln1701* and p.Arg1931* truncating variants using the patient-derived *FANCM*^*−*/*−*^ EGF280 cell line. **a** Western blot showing the FANCM expression in EGF280 cells complemented with lentiviral vectors harboring the three different variants. Bands corresponding to truncated FANCM protein were visible for EGF280 + p.Gln1701* and p.Arg1931*, and no bands were present for the EGF280 + p.Arg658*. **b** Study of the expression of the FANCM protein in EGF280 + p.Arg658*. The c.1972C > T base substitution, causing the p.Arg658* variant abrogates a digestion site for the restriction enzyme *Tse*I that is present in the wild-type (wt) cDNA sequence. Total RNA was extracted from EGF280 + wt*FANCM* and from the EGF280 + p.Arg658* and subjected to reverse transcription. PCR-amplified cDNA products were digested with *Tse*I. Digested and undigested cDNAs were loaded. In the first two lanes are shown bands of 386 bp corresponding to uncut wt cDNA, and bands of 257 and 129 bp corresponding to cut wt cDNA. In next two lanes bands of 386 bp indicate that p.Arg658* cDNA was not cut due to the c.1972C > T base substitution abrogating the *Tse*I site. In the two lanes after the molecular weight marker (M) undigested and digested products of the two previous PCR products were mixed 1:1 and loaded as a control. **c** Analysis of diepoxybutane (DEB) sensitivity on cell survival. The EGF280 cells expressing p.Arg658* are significantly more sensitive to DEB than the cells expressing p.Gln1701* or p.Arg1931* (*P-values* from Tukey’s range test are reported in Supplementary Table 4). EGF280 and EGF280 + wtFANCM are used as controls (*N* = 3; error bars: standard deviation). **d** Chromosome fragility induced by DEB treatment (100 ng/ml). Here, the chromatidic break patterns of the cells expressing wt FANCM, of the cells harboring p.Gln1701* or p.Arg1931* variants, and of the native EGF280 cells or the cells expressing p.Arg658* were statistically different. (*P-values* from chi-squared test; *N* = 2). **e** Analysis of cellular sensitivity to olaparib. Contrarily to what we observed in the DEB sensitivity assays, survival rates of the different complemented cell lines were apparently not different. Human fibroblasts (*BRCA2*^*−*/*−*^) were homozygous for the c.469 A > T (p.Lys157*) truncating variant and were used as a positive control. (*P-values* from Tukey’s range test are reported in Supplementary Table 5; *N* = 3; error bars: standard deviation). All blots derive from the same experiment and were processed in parallel

We tested the functional effect of *FANCM*:p.Arg658*, p.Gln1701*, and p.Arg1931* on DNA repair using genetic complementation assays (Fig. 1). These assays were based on the EGF280 cell line derived from immortalized fibroblasts from a patient who lacked the FANCM protein due to a homozygous c.1506_1507insTA (p.Ile503*, rs764743944) truncating variant.^8^ Complemented *FANCM*^*−/−*^ cells were tested for sensitivity to DEB and olaparib by measuring cell survival and chromosome fragility. The FANCM protein was not detectable in the EGF280 fibroblasts. The transduction of these cells with lentiviral vectors carrying wild-type (wt) FANCM cDNA and cDNAs harboring *FANCM*:p.Gln1701* and p.Arg1931* variants produced, as expected, different C-terminal truncated forms of FANCM. In the EGF280 cells transduced with *FANCM*:p.Arg658* no visible band was observed on western blot (Fig. 1a and Supplementary Fig. 1). As we lack information on the epitope recognized by the antibody, we could not determine whether the p.Arg658*-derived truncated protein was unstable or if the epitope was lost due to the truncation. We therefore analyzed the mRNA expression of *FANCM*:p.Arg658* by reverse transcription and digestion of the PCR-amplified cDNAs. The c.1972C > T base substitution causing the p.Arg658* variant was expected to abolish a digestion site for the restriction enzyme *Tse*I present in the wt sequence. *Tse*I-digestion of wt and mutated cDNAs clearly indicated the presence of a mutated mRNA product in the EGF280 cells transduced with *FANCM*:p.Arg658* (Fig. 1b and Supplementary Fig. 1).

In the DEB sensitivity-based assay (Fig. 1c), the EGF280 patient-derived cell line showed a high-sensitivity phenotype, that was rescued by expression of the wt *FANCM*. EGF280 cells expressing *FANCM*:p.Arg658* failed to rescue DEB sensitivity and showed survival rates overlapping with those of the native EGF280 cells. In comparison, cells expressing *FANCM*:p.Gln1701* and p.Arg1931* variants showed an intermediate phenotype with survival rates significantly higher than those of EGF280 cells, though significantly lower than those of the cells expressing wt FANCM (Fig. 1c and Supplementary Table 2). These results were confirmed in the chromosome fragility tests where the number of chromatidic breaks in cells harboring p.Gln1701* or p.Arg1931* variants was statistically lower than that of EGF280 cells or cells expressing the p.Arg658* and statistically higher than that of cells expressing wt FANCM (Fig. 1d). In the olaparib sensitivity-based assay, the survival rates of the cell lines transduced with the three FANCM truncating variants were not statistically different. Only at higher olaparib concentrations (>5000 nM) the survival rates of these cell lines were significantly lower than that of the wt FANCM cells and higher than that of the EGF280 cells (Fig. 1e and Supplementary Table 3).

## Discussion

In this study we investigated the association of the three recurrent *FANCM* truncating variants p.Arg658*, p.Gln1701*, and p.Arg1931*, with breast cancer risk overall and by tumor subtype. While in non-Finnish Europeans these are the three most common *FANCM* truncating variants, their carrier frequency is low being 0.033, 0.21 and 0.21%, respectively (https://gnomad.broadinstitute.org/).^15^ We conducted large case-control studies in 67,112 unselected breast cancer cases, 53,766 controls, and 26,662 carriers of *BRCA1* or *BRCA2* pathogenic variants. Furthermore, we performed functional analyses based on a patient-derived *FANCM*^*−/−*^ cell line transduced with vectors carrying the three *FANCM* variants and tested for sensitivity to DEB or olaparib. Our genetic data suggest that *FANCM*:p.Arg658* is a risk factor for ER-negative and TNBC subtypes with statistically significant ORs of 2.44 and 3.79, respectively. These associations were confirmed when we restricted the analyses to countries with higher carrier frequencies. In these restricted analyses we also found that the p.Arg1931* was associated with breast cancer risk in the ER-negative subtype with statistically significant OR = 1.96. (Table 1). These data, together with previously published genetic studies,^10–13^ confirm that *FANCM* truncating variants are risk factors for breast cancer, with a stronger association for the ER-negative and TNBC subtypes. Our functional data, obtained in a background of a *FANCM* null cell line, support these findings showing that all three truncating variants were deleterious; hence, it is expected that, in the heterozygous state, any of these *FANCM* variants have partial activity. In the functional tests, we also observed that olaparib had a greater effect on survival of the cells harboring any of the *FANCM*:p.Arg658*, p.Gln1701*, or p.Arg1931* variants with respect to that on EGF280 cells complemented with wt FANCM (Fig. 1e). As this is consistent with previous results,^16^ PARP1 inhibition might be a possible therapeutic approach to treat patients with breast tumors associated with germline *FANCM* pathogenic variants. On the contrary, the DEB sensitivity assays showed that *FANCM*:p.Arg658*, is associated with a stronger impairment of DNA repair activity, compared to p.Gln1701* and p.Arg1931*, possibly reflecting the position of protein truncation (Fig. 1c, d).

**Fig. 2:**
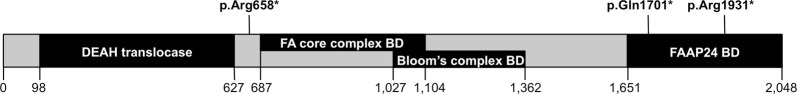
Schematic diagram of the 2,048 amino acid long FANCM protein. The functional or binding domains (BD) are indicated in black and as reported in Deans and West, 2009. The position of the three *FANCM* truncating variants c.1972C > T (p.Arg658*), c.5101 C > T (p.Gln1701*) and c.5791 C > T (p.Arg1931*) is also shown

*FANCM* encodes for a key protein of the upstream FA/BRCA pathway mediating the assembly of the FA core complex. This protein is 2048 AA long, possesses in its N-terminal region an intrinsic ATP-dependent DNA translocase activity and, with its central region, recognizes the Bloom’s complex, which is also involved in the DNA HR repair. By interacting with its C-terminal binding partner, the FA associated protein 24 (FAAP24), the FANCM protein brings to sites of ICL DNA lesions the FA and the Bloom’s complexes initiating HR repair^17^ (Fig. 2). We studied FANCM:p.Arg658*, p.Gln1701*, and p.Arg1931* in the same genetic *FANCM*^*−/−*^ background and showed that, after exposure to DEB, the N-terminal *FANCM*:p.Arg658* had a statistically stronger effect on cell survival and chromosome stability (presumably due to less efficient DNA repair activity) than did p.Gln1701* and p.Arg1931*. This also suggests that in human living cells the *FANCM*:p.Arg658* variant might impair DNA repair more severely than p.Gln1701* and p.Arg1931*. We have shown that in vitro both the p.Gln1701*- and the p.Arg1931*-derived FANCM proteins are expressed and that the p.Arg658*-mRNA is transcribed (Fig. 1a, b). An N-terminus fragment including the first 422 AA of FANCM was shown to be stable when expressed in human cell lines,^17^ thus supporting the possibility that the *FANCM*:p.Arg658*-derived protein may also be expressed and stable. Hence, we hypothesize that the observed difference in survival and chromosome fragility of cells treated with DEB may be attributable to the diverse residual function of the different truncated forms of FANCM. In fact, the p.Gln1701*- and the p.Arg1931*-derived forms are expected to lose the interaction with FAAP24, but to retain the ability of binding other FANCM interacting proteins. Hence, our data suggest that the lack of interaction between FANCM and FAAP24 has a less severe impact on the DNA damage response than when protein truncation occurs upstream the FANCM domains AA 687–1104 and AA 1027–1362 mediating the interaction with the FA core complex and the Bloom’s complex, respectively.

Previously published genetic and clinical data support our hypothesis of a position effect. *FANCM* pathogenic variants were shown to be associated with a moderate risk of developing high-grade serous epithelial ovarian cancer, but p.Arg1931* appeared to confer a lower risk.^18^ Moreover, five female breast cancer probands carrying homozygous *FANCM* truncating variants were recently described.^9^ Three of these, two homozygous for p.Gln1701*, and one for p.Arg1931*, developed breast cancer at age 52 years or later and their cells did not demonstrate chromosome fragility. The other two probands were homozygous for p.Arg658* and developed early-onset breast cancer (at age 29 and 32); in addition, one developed several cancers, and the other demonstrated chromosomal fragility.^9^

Due to the rarity of the studied mutations in most populations, estimation of the risks is challenging. Preferably, the cases should be examined in comparison to geographically, ethnically and genetically matched controls. In the Finnish population, p.Gln1701* and p.Arg1931* are reported with carrier frequency of 1.62% and of 0.92%, respectively (https://gnomad.broadinstitute.org/).^15^ Case-control studies based on the Finnish population showed a strong statistical evidence of association of p.Gln1701* with ER-negative disease, with OR of 2.37 (95% CI = 1.37–4.12, *P* = 0.0021), and with TNBC with ORs of 3.56 (95% CI = 1.81–6.98, *P* = 0.0002),^10^ while p.Arg1931* was found associated with TNBC with an OR of 5.14 (95% CI = 1.65–16.0, *P* = 0.005).^12^ However, as our 95% CI of risk estimates for TNBC included odds ratios of 2 for both the latter mutations, the published and our results are not mutually exclusive. Risk estimates associated with rare variants may depend on their frequency and the genetic background of the population studied. Hence, pooling the data from multiple outbred and admixed populations as it was done in the present study, may yield different risk estimates than those derived from geographically, ethnically and genetically matched controls, as in the Finnish studies. Indeed, it would have been interesting to test the *FANCM* variant position effect in the Finnish population, but unfortunately the p.Arg658* is very rare if not absent in this population (https://gnomad.broadinstitute.org/).^15^

Recent attempts to identify novel, high- to moderate-risk breast cancer-predisposing genes have not been particularly fruitful. However, a few genes have emerged as potential risk factors for ER-negative disease and TNBC, with *FANCM*, *BRIP1*, and *RAD51C* being among those suggested to confer moderate risk of these subtypes. Other predisposing genes increasing the risk of ER-negative and TNBC may also exist. Hence, further gene discovery efforts should take into consideration that risk-associated variants may be associated with specific tumor subtypes and/or variation in risk may depend on the variant position. In addition, we provide evidence that lack of FANCM protein and truncating variants identified in breast cancer patients are associated with increased sensitivity to the PARPi olaparib suggesting a therapeutic opportunity to treat FANCM-associated breast tumors that warrants further investigation. The PARPi sensitivity test may also prove useful for preclinical investigation of further truncating or missense *FANCM* variants.

In summary, we have shown that *FANCM*:p.Arg658* is associated with risk of ER-negative breast cancer and TNBC. The outcomes of functional assays testing the DNA repair efficiency in complemented human cells support the hypothesis that breast cancer risk may be greater for N-terminal than C-terminal *FANCM* truncating variants. Further genetic studies and meta-analyses are warranted to derive more precise risk estimates for the different *FANCM* variants.

## Methods

### Study participants

The individuals included in this study were women of genetically confirmed European ancestry who were originally ascertained in 73 case-control studies from 19 countries participating in the BCAC or in 59 studies enrolling *BRCA1* or *BRCA2* pathogenic variants carrier from 30 countries participating in the CIMBA.

### Ethics

All participating studies, listed in Supplementary Table 4 and Supplementary Table 5, were approved by their ethics review boards and followed national guidelines for informed consent. However, due to the retrospective nature of the majority of the studies, not all participant individuals have provided written informed consent to take part in the present analysis. The Milan Breast Cancer Study Group (MBCSG) was approved by ethics committee from Istituto Nazionale dei Tumori di Milano and Istituto Europeo di Oncologia, in Milan.

The BCAC studies contributed 67,112 invasive breast cancer cases and 53,766 controls. The majority of these studies were population-based, hospital-based or case-control studies nested within population-based cohorts (86%); few were family-clinic-based studies (14%; Supplementary Table 4). For each study subject, information on the disease status and the age at diagnosis or at interview were provided. Data on lifestyle risk factors were available for most subjects and clinical and pathological data were available for most cases. All these data were incorporated in the BCAC dataset (version 10). A total of 44,565 (66%) cases were ER-positive, 10,770 (16%) were ER-negative, and 4,805 (7%) were TNBC; 13,743 (20%) had a positive first-degree family history of breast cancer.

The CIMBA studies contributed 15,679 carriers of a pathogenic *BRCA1* variant and 10,983 carriers of a pathogenic *BRCA2* variant to this analysis (Supplementary Table 5). Nearly all (98%) of these carriers were ascertained through cancer genetic clinics; few carriers were recruited by population-based sampling of cases or by community recruitment. In some instances, multiple members of the same family were included. For each pathogenic variant carrier, the information on the type of the *BRCA1* or *BRCA2* variant, disease status, and censoring variables (see below, *Statistical analyses*) were collected and included in the CIMBA database.

### Genotyping

Genotyping of *FANCM*:p.Arg658*, p.Gln1701*, and p.Arg1931* truncating variants was conducted using a custom-designed Illumina genotyping array (the “OncoArray”, Illumina, Inc. San Diego, CA, USA) at six independent laboratories. To ensure consistency of the genotype data, all laboratories used the same genotype-clustering file and genotyped the same set of reference-samples selected from the HapMap project. Samples with a call rate <95% and those with heterozygosity <5% or >40% were excluded. Further details of the genotype-calling and quality control have been described previously.^14^ The cluster plots of the three *FANCM* truncating variants were curated manually to confirm the automatic calls (Supplementary Fig. 2).

### Statistical analyses

The BCAC data were analyzed to test the association between *FANCM*:p.Arg658*, p.Gln1701*, and p.Arg1931* and breast cancer risk. Logistic regression analyses were performed to estimate ORs with 95% CIs for variant carriers versus non-carriers, adjusting for country and the first ten principal components, as previously described.^19^ *P-*values were calculated by applying the likelihood ratio test (LRT) comparing the model containing the variant carrier status as a covariate to a model without the variant carrier status. The primary analyses were performed including all invasive breast cancer cases and controls and subgrouping cases based on tumor hormonal status. We then performed a country-restricted analysis including the 50% of the countries with the higher variant carrier frequencies. Specifically, we included only countries in which the carrier frequencies in cases and controls combined were higher than the median of the carrier frequencies observed in all countries. Median frequencies were 0.007, 0.114 and 0.163 for p.Arg658*, p.Gln1701* and p.Arg1931* carriers, respectively.

The CIMBA data were analyzed to evaluate the association between each *FANCM* truncating variant and breast cancer risk in carriers of *BRCA1* or *BRCA2* pathogenic variant. A survival analyses framework was applied. Briefly, each variant carrier was followed from the age of 18 years until the first breast cancer diagnosis, or censored as unaffected at ovarian cancer diagnosis, bilateral prophylactic mastectomy, or age at last follow-up. The analyses were performed by modelling the retrospective likelihood of the observed genotypes conditional on the disease phenotype as detailed previously.^20^ All analyses were stratified for country. The per-allele hazard ratio (HR), 95% CIs were estimated separately for each variant. A score test was used to derive *P-*values for the associations. The analyses of the BCAC data were performed using STATA version 15 (StataCorp LLC, College Station, Texas, USA). The analyses of the CIMBA data were carried out using custom-written code in Python and Fortran. All statistical tests were two-sided and *P-*values <0.05 were considered statistically significant.

### Cell lines, plasmids, and lentiviral particles production and transduction

The immortalized patient-derived *FANCM*^*−/−*^ cell line EGF280^8^ was transduced with pLenti CMV rtTA3 Blast, a gift from E. Campeau (Addgene plasmid #26429). The doxycycline-inducible lentiviral vector pLVX-TRE3G-FANCM, a gift from N. Ameziane (Vrije Universiteit Medical Center, Amsterdam) was mutated by site-directed mutagenesis using the QuickChange II XL Site-Directed Mutagenesis Kit (Agilent Technologies) and the following PAGE purified mutagenic primers. *FANCM* c.1972C > T primer 1: 5’-GCCTTCTCGGAACTTGCAGTGAAAGTCATCTATCTTTTCC-3’ and primer 2: 5’-GGAAAAGATAGATGACTTTCACTGCAAGTTCCGAGAAGGC-3’ for the p.Arg658*; *FANCM* c.5101 C > T primer 1: 5’-TTAAACAATGGTCCTATTGTTTGTTCTTCTTAACAGTGCTTGGGT-3’ and primer 2: 5’-ACCCAAGCACTGTTAAGAAGAACAAACAATAGGACCATTGTTTAA-3’ for the p.Gln1701*. Generation of the lentiviral vector containing the *FANCM*:c.5791 C > T (p.Arg1931*) and transduction of the EGF280 cells were already described.^11^ Expression of exogenous FANCM protein was achieved supplementing cell culture medium with doxycycline (1 μg/ml, final concentration). All the cell lines used in this study were routinely checked for mycoplasma contamination using the MycoAlert™ Mycoplasma Detection Kit (Lonza).

### Western blot and mRNA expression studies

Cell lysis and western blot assays were performed as previously described.^8^ The following primary antibodies were used: mouse monoclonal anti-FANCM antibody, clone CV5.1 diluted 1:100 (ref: MABC545, MERCK Millipore), mouse monoclonal anti-Vinculin diluted 1:3000 (ref: ab18058, abcam). Western blotting detection was achieved with Luminata^TM^ Classico (Millipore) (Vinculin) and LuminataForte^TM^ (Millipore) (FANCM). We used RT PCR to test the expression of the mutant *FANCM*:p.Arg658*. Total RNA was extracted (RNeasy Mini Kit Qiagen) from the wt*FANCM* and *FANCM*:p.Arg658* transduced EGF280 cell lines. Reverse transcription was performed using High-Capacity RNA-to-cDNA Kit (Thermofisher); a cDNA region corresponding to the FANCM sequence containing the amino acid (AA) position Arg658 was amplified by PCR using the forward: 5’-AGTAACAGGCAGGTCCTTCA-3´and reverse: 5’-TGATCTTGCCACAGTCTCCA-3’ primers. The 386 bp PCR products were then digested with *Tse*I restriction enzyme (New England Biolabs) for two hours at 65 °C and analyzed by standard agarose gel electrophoresis.

### Cell survival assay

The effect of the different *FANCM* variants on cell survival was measured with a Sulforhodamine B (SRB) assay.^21^ One-thousand cells were seeded in 96-well plates and treated constantly with DEB or PARPi olaparib at the indicated concentrations until untreated cells reached confluency. Cell monolayers were fixed overnight at 4 °C with 75 μl of 20% trichloroacetic acid (TCA). TCA was aspirated, and cells washed with tap water. Once dried, 50 μl of SRB was added to the wells and plates were incubated on a shaker at room temperature for 30 min. The excess of SRB dye was removed by washing repeatedly with 1% acetic acid, the plates were dried for 20 min, and the protein-bound dye was dissolved in 10 mM Tris for OD determination at 492 nm using a microplate reader (Tecan Sunrise™, Tecan Group Ltd. Männedorf, Switzerland). At least three independent experiments were performed for each cell line and in each experiment, 12 wells were measured per concentration point. These results were statistically analyzed using the Prism (GraphPad) software. Two-Way ANOVA test was used for single comparisons between different cell lines and statistical significance was assessed with the Tukey’s range test. A *P-*value < 0.05 was considered statistically significant.

### Chromosome fragility test

Chromosome fragility test was performed as previously described.^11^ Twenty-five metaphases were scored for chromosome breakages using the Metafer Slide Scanning Platform from Metasystems. Results were graphed as distributions of metaphases presenting 0, 1, and >1 chromatid break. Statistical analysis was performed applying chi-squared test.

### Reporting summary

Further information on research design is available in the Nature Research Reporting Summary

## Supplementary information

Supplementary material

Reporting Summary Checklist

## Data Availability

A subset of the genotype data analysed in this study is publicly available from the dbGaP repository and can be accessed at https://identifiers.org/dbgap:phs001265.v1.p1 (data generated as part of the BCAC studies) and at https://identifiers.org/dbgap:phs001321.v1.p1 (data generated as part of the CIMBA studies). The remaining genotype data analysed in this study (and generated as part of the BCAC and CIMBA studies listed in Supplementary Tables 4 and 5 of the related article, respectively) are not publicly available due to restraints imposed by the ethics committees of individual studies, but can be accessed from the corresponding author on reasonable request as described at 10.6084/m9.figshare.8982296.^22^ Additional datasets generated during this study (and supporting Fig. 1 and Supplementary Tables 2 and 3 in the published article) are available on request as described above. The data generated and analyzed during this study are described in the following data record: 10.6084/m9.figshare.8982296.^22^
