# Supplementary material for: The FANCM:p.Arg658* truncating variant is associated with risk of triple-negative breast cancer
Source: NPJ Breast Cancer. 2019 Nov 1;5:38. doi: 10.1038/s41523-019-0127-5 (PMC6825205; doi:10.1038/s41523-019-0127-5)

Supplementary material

The *FANCM*:p.Arg658* truncating variant is associated with risk of triple-negative breast cancer

Figlioli *et al.*

Supplementary Table 1. Association analysis of the *FANCM*:p.Arg658*, p.Gln1701*, p.Arg1931* truncating variants in carriers of pathogenic *BRCA1* or *BRCA2* variants.

	BRCA1						***BRCA2***					
**Subgroup**	**Carriers**	**Non-carriers**	**Freq %**	**HR[a]**	**95% CI**	***P*[b]**	**Carriers**	**Non-carriers**	**Freq %**	**HR[a]**	**95% CI**	***P*[b]**
FANCM*:p.Arg658												
Unaffected	1	7,784	0.013	-			1	5,367	0.019	-		
Affected	5	7,870	0.063	2.40	0.52-11.12	0.262	4	5,598	0.071	2.13	0.41-11.14	0.369
FANCM*:p.Gln1701												
Unaffected	16	7,774	0.205	-			7	5,368	0.13	-		
Affected	12	7,876	0.152	0.66	0.32-1.33	0.253	11	5,597	0.196	1.19	0.49-2.85	0.699
FANCM*:p.Arg1931												
Unaffected	16	7,765	0.206	-			3	5,371	0.056	-		
Affected	11	7,870	0.139	0.71	0.36-1.43	0.34	5	5,602	0.089	1.27	0.33-4.88	0.728

Freq, frequency; HR, hazard ratio; CI, confidence interval. [a] Stratified for country; [b] P-value from score test.

Supplementary Table 2. Two-Way ANOVA test (Tukey's range test) for single comparisons between different cell lines in the DEB sensitivity assay.

**Group comparison**	*P*
**[DEB] = 25 ng/ml**	
EGF280 + wtFANCM vs. EGF280 KO	****
EGF280 + wtFANCM vs. EGF280 + p.Arg1931*	ns
EGF280 + wtFANCM vs. EGF280 + p.Gln1701*	ns
EGF280 + wtFANCM vs. EGF280 + p.Arg658*	****
EGF280 KO vs. EGF280 + p.Arg1931*	*
EGF280 KO vs. EGF280 + p.Gln1701*	**
EGF280 KO vs. EGF280 + p.Arg658*	ns
EGF280 + p.Arg1931* vs. EGF280 + p.Gln1701*	ns
EGF280 + p.Arg1931* vs. EGF280 + p.Arg658*	ns
EGF280 + p.Gln1701* vs. EGF280 + p.Arg658*	ns
**[DEB] = 50 ng/ml**	
EGF280 + wtFANCM vs. EGF280 KO	****
EGF280 + wtFANCM vs. EGF280 + p.Arg1931*	*
EGF280 + wtFANCM vs. EGF280 + p.Gln1701*	**
EGF280 + wtFANCM vs. EGF280 + p.Arg658*	****
EGF280 KO vs. EGF280 + p.Arg1931*	****
EGF280 KO vs. EGF280 + p.Gln1701*	****
EGF280 KO vs. EGF280 + p.Arg658*	ns
EGF280 + p.Arg1931* vs. EGF280 + p.Gln1701*	ns
EGF280 + p.Arg1931* vs. EGF280 + p.Arg658*	****
EGF280 + p.Gln1701* vs. EGF280 + p.Arg658*	****
**[DEB] = 100 ng/ml**	
EGF280 + wtFANCM vs. EGF280 KO	****
EGF280 + wtFANCM vs. EGF280 + p.Arg1931*	**
EGF280 + wtFANCM vs. EGF280 + p.Gln1701*	*

EGF280 + wtFANCM vs. EGF280 + p.Arg658*	****
EGF280 KO vs. EGF280 + p.Arg1931*	****
EGF280 KO vs. EGF280 + p.Gln1701*	****
EGF280 KO vs. EGF280 + p.Arg658*	ns
EGF280 + p.Arg1931* vs. EGF280 + p.Gln1701*	ns
EGF280 + p.Arg1931* vs. EGF280 + p.Arg658*	****
EGF280 + p.Gln1701* vs. EGF280 + p.Arg658*	****
**[DEB] = 200 ng/ml**	
EGF280 + wtFANCM vs. EGF280 KO	****
EGF280 + wtFANCM vs. EGF280 + p.Arg1931*	**
EGF280 + wtFANCM vs. EGF280 + p.Gln1701*	*
EGF280 + wtFANCM vs. EGF280 + p.Arg658*	****
EGF280 KO vs. EGF280 + p.Arg1931*	****
EGF280 KO vs. EGF280 + p.Gln1701*	****
EGF280 KO vs. EGF280 + p.Arg658*	ns
EGF280 + p.Arg1931* vs. EGF280 + p.Gln1701*	ns
EGF280 + p.Arg1931* vs. EGF280 + p.Arg658*	****
EGF280 + p.Gln1701* vs. EGF280 + p.Arg658*	****

Legend. *P*, *P-value*; ns, not significant; *, *P*<0,05; **, *P*<0,01; ***, *P*<0,001; ****, *P*<0,0001.

Supplementary Table 3. Two-Way ANOVA test (Tukey's range test) for single comparisons between different cell lines in the olaparib sensitivity assay.

Group comparison	*P*
**[Olaparib] = 5 nM**	
EGF280 + wtFANCM vs. EGF280 KO	ns
EGF280 + wtFANCM vs. EGF280 + p.Arg1931*	ns
EGF280 + wtFANCM vs. EGF280 + p.Arg658*	ns
EGF280 + wtFANCM vs. EGF280 + p.Gln1701*	ns
EGF280 + wtFANCM vs. BRCA2	ns
EGF280 KO vs. EGF280 + p.Arg1931*	ns
EGF280 KO vs. EGF280 + p.Arg658*	ns
EGF280 KO vs. EGF280 + p.Gln1701*	ns
EGF280 KO vs. BRCA2	ns
EGF280 + p.Arg1931* vs. EGF280 + p.Arg658*	ns
EGF280 + p.Arg1931* vs. EGF280 + p.Gln1701*	ns
EGF280 + p.Arg1931* vs. BRCA2	ns
EGF280 + p.Arg658* vs. EGF280 + p.Gln1701*	ns
EGF280 + p.Arg658* vs. BRCA2	ns
EGF280 + p.Gln1701* vs. BRCA2	ns
**[Olaparib] = 50 nM**	
EGF280 + wtFANCM vs. EGF280 KO	**
EGF280 + wtFANCM vs. EGF280 + p.Arg1931*	ns
EGF280 + wtFANCM vs. EGF280 + p.Arg658*	ns
EGF280 + wtFANCM vs. EGF280 + p.Gln1701*	ns
EGF280 + wtFANCM vs. BRCA2	ns
EGF280 KO vs. EGF280 + p.Arg1931*	*
EGF280 KO vs. EGF280 + p.Arg658*	ns

EGF280 KO vs. EGF280 + p.Gln1701*	**
EGF280 KO vs. BRCA2	ns
EGF280 + p.Arg1931* vs. EGF280 + p.Arg658*	ns
EGF280 + p.Arg1931* vs. EGF280 + p.Gln1701*	ns
EGF280 + p.Arg1931* vs. BRCA2	ns
EGF280 + p.Arg658* vs. EGF280 + p.Gln1701*	ns
EGF280 + p.Arg658* vs. BRCA2	ns
EGF280 + p.Gln1701* vs. BRCA2	ns
**[Olaparib] = 500 nM**	
EGF280 + wtFANCM vs. EGF280 KO	****
EGF280 + wtFANCM vs. EGF280 + p.Arg1931*	ns
EGF280 + wtFANCM vs. EGF280 + p.Arg658*	**
EGF280 + wtFANCM vs. EGF280 + p.Gln1701*	ns
EGF280 + wtFANCM vs. BRCA2	****
EGF280 KO vs. EGF280 + p.Arg1931*	**
EGF280 KO vs. EGF280 + p.Arg658*	ns
EGF280 KO vs. EGF280 + p.Gln1701*	****
EGF280 KO vs. BRCA2	ns
EGF280 + p.Arg1931* vs. EGF280 + p.Arg658*	ns
EGF280 + p.Arg1931* vs. EGF280 + p.Gln1701*	ns
EGF280 + p.Arg1931* vs. BRCA2	****
EGF280 + p.Arg658* vs. EGF280 + p.Gln1701*	**
EGF280 + p.Arg658* vs. BRCA2	**
EGF280 + p.Gln1701* vs. BRCA2	****
**[Olaparib] = 5000 nM**	
EGF280 + wtFANCM vs. EGF280 KO	****
EGF280 + wtFANCM vs. EGF280 + p.Arg1931*	**
EGF280 + wtFANCM vs. EGF280 + p.Arg658*	****
EGF280 + wtFANCM vs. EGF280 + p.Gln1701*	*
EGF280 + wtFANCM vs. BRCA2	****
EGF280 KO vs. EGF280 + p.Arg1931*	*
EGF280 KO vs. EGF280 + p.Arg658*	ns
EGF280 KO vs. EGF280 + p.Gln1701*	***
EGF280 KO vs. BRCA2	ns
EGF280 + p.Arg1931* vs. EGF280 + p.Arg658*	ns
EGF280 + p.Arg1931* vs. EGF280 + p.Gln1701*	ns
EGF280 + p.Arg1931* vs. BRCA2	***
EGF280 + p.Arg658* vs. EGF280 + p.Gln1701*	ns
EGF280 + p.Arg658* vs. BRCA2	*
EGF280 + p.Gln1701* vs. BRCA2	****

Legend. *P*, *P-value*; ns, not significant; *, $P<0{,}05$; **, $P<0{,}01$; ***, $P<0{,}001$; ****, $P<0{,}0001$.

Supplementary Table 4. Description of the BCAC studies included in the present analysis.

Study name	Study acronym	Country	Study design	Controls	Cases
The Two Sister Study	2SISTER	USA	Cases from sister-matched case-control study	0	919
Australian Breast Cancer Family Study	ABCFS	Australia	Population-based case-control study	187	1,117
Amsterdam Breast Cancer Study	ABCS	Netherlands	Hospital-based consecutive cases; population-based controls	189	347
Australian Breast Cancer Tissue Bank	ABCTB	Australia	Hospital-based multi-site newly diagnosed breast cancer case	375	947
Agricultural Health Study	AHS	USA	Prospective cohort study: nested case-control	1,137	513
Bavarian Breast Cancer Cases and Controls	BBCC	Germany	Hospital-based cases; population-based controls	253	403
British Breast Cancer Study	BBCS	UK	Cancer registry and National Cancer Research network (NCRN) based cases; population-based controls	442	122
Breast Cancer Employment and Environment Study	BCEES	Australia	Population-based case-control study	835	783
New York Breast Cancer Family Registry	BCFR-NY	USA	Clinic-based recruitment of families; family-based cohort	27	401
Philadelphia Breast Cancer Family Registry	BCFR-PA	USA	Clinic-based recruitment of families; family-based cohort	0	67
Utah Breast Cancer Family Registry	BCFR-UT	USA	Clinic-based recruitment of non-BRCA1/2 familial breast cancer cases; unaffected BRCA1/2 carriers as controls	0	101
Breast Cancer in Northern Israel Study	BCINIS	Israel	Population-based case-control study	724	1,337
Breast Oncology Galicia Network	BREOGAN	Spain	Population-based case-control	725	1,266
Breast Cancer Study of the University of Heidelberg	BSUCH	Germany	Hospital-based cases; healthy blood donator controls	168	252
Canadian Breast Cancer Study	CBCS	Canada	Population-based case-control study	817	568
Crete Cancer Genetics Program	CCGP	Greece	Hospital-based case-control study	332	667
CECILE Breast Cancer Study	CECILE	France	Population-based case-control study	159	280
Copenhagen General Population Study	CGPS	Denmark	Population-based case-control study	716	1,408
Cancer Prevention Study-II Nutrition Cohort	CPSII	USA	Nested case-control study	3,028	2,388
California Teachers Study	CTS	USA	Prospective cohort study: nested case-control	610	1,156
DietCompLyf Breast Cancer Survival Study	DIETCOMPLYF	UK	Multi-center prospective cohort study	0	708
European Prospective Investigation Into Cancer and Nutrition	EPIC	France, Germany, Greece, Italy, Spain, The Netherlands, and UK	Case-control study, nested in a prospective cohort study	3,644	3,435
ESTHER Breast Cancer Study	ESTHER	Germany	Population-based case-control study	187	291
Family History Risk Study	FHRISK	UK	Clinic-based cohort study with a nested case-control study	296	102
German Consortium for Hereditary Breast & Ovarian Cancer	GC-HBOC	Germany	Clinic-based familial case-control study	1,593	3,416
Gene Environment Interaction and Breast Cancer in Germany	GENICA	Germany	Population-based case-control study	284	459
A randomized phase II trial investigating the addition of carboplatin to neoadjuvant therapy for triple-negative and HER2-positive early breast cancer	GEPARSIXTO	Germany	Multicenter, prospective, randomized, open-label phase II study	0	387
Genetic Epidemiology Study of Breast Cancer by Age 50	GESBC	Germany	Population-based study of women <50 years	181	312
Hannover Breast Cancer Study	HABCS	Germany	Hospital-based case-control study	866	909
Hospital Clinico San Carlos	HCSC	Spain	Population-based study of priori sporadic breast cancer cases	0	423

Helsinki Breast Cancer Study	HEBCS	Finland	Hospital-based case-control study, plus additional familial cases	177	281
Hannover-Minsk Breast Cancer Study	HMBCS	Belarus	Hospital-based cases; population-based controls	249	212
Hannover-Ufa Breast Cancer Study	HUBCS	Russia	Hospital-based cases; population-based controls	120	211
Karolinska Breast Cancer Study	KARBAC	Sweden	Hospital-based cases; population-based controls	0	500
Karolinska Mammography Project for Risk Prediction of Breast Cancer - Cohort Study	KARMA	Sweden	Nested case-control study within a population-based cohort, plus prevalent cases	6,026	2,366
Kuopio Breast Cancer Project	KBCP	Finland	Clinic-based case; population-based controls	245	522
Leuven Multidisciplinary Breast Centre	LMBC	Belgium	Hospital-based case-control study	1,268	784
Macedonian Breast Cancer Study	MABCS	Macedonia	Hospital-based case-control study	92	89
Mammary Carcinoma Risk Factor Investigation	MARIE	Germany	Population-based case-control study	289	506
Milan Breast Cancer Study Group	MBCSG	Italy	Clinic-based recruitment of familial/early onset breast cancer cases; population-based controls	366	549
Mayo Clinic Breast Cancer Study	MCBCS	USA	Hospital-based case-control study	221	749
Melbourne Collaborative Cohort Study	MCCS	Australia	Prospective cohort study: nested case-control study	978	870
Multiethnic Cohort	MEC	USA	Prospective cohort study: nested case-control	724	668
Melanoma Inquiry of Southern Sweden	MISS	Sweden	Nested case-control study within a population-based prospective cohort	1,545	599
Mayo Mammography Health Study	MMHS	USA	Nested case-control study within a prospective cohort study	1,635	275
Memorial Sloan-Kettering Cancer Center Study	MSKCC	USA	Case-control study	0	136
Montreal Gene-Environment Breast Cancer Study	MTLGEBCS	Canada	Population-based case-control study	170	341
Nashville Breast Health Study	NBHS	USA	Population-based case-control study	652	483
Northern California Breast Cancer Family Registry	NC-BCFR	USA	Population-based familial case-control study	151	759
North Carolina Breast Cancer Study	NCBCS	USA	Population-based case-control study	1,006	2,074
Nurses Health Study	NHS	USA	Prospective cohort study: nested case-control	1,804	1,103
Nurses Health Study 2	NHS2	USA	Nested case-control study within a population-based cohort	1,905	1,112
Ontario Familial Breast Cancer Registry	OFBCR	Canada	Population-based familial case-control study	375	1,662
Leiden University Medical Centre Breast Cancer Study	ORIGO	Netherlands	Hospital-based prospective cohort study	660	921
NCI Polish Breast Cancer Study	PBCS	Poland	Population-based case-control study	2,045	1,740
Karolinska Mammography Project for Risk Prediction of Breast Cancer - Case-Control Study	pKARMA	Sweden	Case-control study	48	740
The Prostate,Lung,Colorectal and Ovarian (PLCO) Cancer Screening Trial	PLCO	USA	Prospective cohort study: nested case-control	2,595	1,822
Prospective Study of Outcomes in Sporadic Versus Hereditary Breast Cancer	POSH	UK	Prospective cohort	0	1,088
Evaluation of Predictive Factors regarding the Effectivity of Aromatase Inhibitor Therapy	PREFACE	Germany	Multicenter, prospective, randomized, open-label phase IV study	0	2,950
Predicting the Risk Of Cancer At Screening Study	PROCAS	UK	Population-based study	1,656	342
Rotterdam Breast Cancer Study	RBCS	Netherlands	Hospital-based case-control study	240	450
Study of Epidemiology and Risk factors in Cancer Heredity	SEARCH	UK	Population-based case-control study	2,673	4,057
The Sister Study	SISTER	USA	Case-cohort nested in prospective cohort of women with a sister-history of breast cancer	1,562	1,502
Städtisches Klinikum Karlsruhe Deutsches Krebsforschungszentrum Study	SKKDKFZS	Germany	Hospital-based case cohort	0	1,086
Swedish Mammography Cohort	SMC	Sweden	Nested case control study within a population-based cohort	704	1,509

Simultaneous Study of Gemcitabine-Docetaxel Combination adjuvant treatment	SUCCESSB	Germany	Multicenter, prospective, randomized, open-label phase III study	0	440
Simultaneous Study of Docetaxel Based Anthracycline Free Adjuvant Treatment Evaluation	SUCCESSC	Germany	Multicenter, prospective, randomized, open-label phase III study	0	2,836
IHCC-Szczecin Breast Cancer Study	SZBCS	Poland	Hospital-based case-control study	174	352
Triple-negative Breast Cancer Consortium	TNBCC	Various	Multiple case series	0	113
UCI Breast Cancer Study	UCIBCS	USA	Population-based case-control study	258	427
Generations Study	UKBGS	UK	Nested case-control study of incident and prevalent cases within prospective cohort study	705	1,048
United Kingdom Ovarian cancer Population Study	UKOPS	UK	Hospital-based ovarian case-control study	974	0
US Radiologic Technologists Study	USRT	USA	Population-based case-control study using prevalent cases with controls matched to cases on year of birth	1,699	1,354
**Total**				**53,766**	**67,112**

Supplementary Table 5. Description of the CIMBA studies included in the present analysis.

			BRCA1		***BRCA2***	
**Study name**	**Study acronym**	**Country**	**Unaffected**	**Breast Cancer**	**Unaffected**	**Breast Cancer**
Australian site of the Breast Cancer Family Registry	BCFR-AU	Australia	14	25	11	28
Northern California site of the Breast Cancer Family Registry	BCFR-NC	USA	4	33	5	22
New York site of the Breast Cancer Family Registry	BCFR-NY	USA	25	37	27	25
Ontario site of the Breast Cancer Family Registry	BCFR-ON	Canada	34	88	24	60
Philadelphia site of the Breast Cancer Family Registry	BCFR-PA	USA	26	18	3	3
Utah site of the Breast Cancer Family Registry	BCFR-UT	USA	135	67	97	54
Baltic Familial Breast Ovarian Cancer Consortium	BFBOCC	Lituania/Latvia	133	111	6	12
Beth Israel Deaconess Medical Center	BIDMC	USA	41	44	28	24
BRCA-gene mutations and breast cancer in South African women	BMBSA	South Africa	21	37	53	87
Beckman Research Institute of the City of Hope	BRICOH	USA	98	52	76	48
Rigshospitalet	CBCS	Denmark	111	76	65	64
Spanish National Cancer Centre	CNIO	Spain	32	31	26	33
City of Hope Cancer Center	COH	USA	84	141	43	98
CONsorzio Studi ITaliani sui Tumori Ereditari Alla Mammella	CONSIT TEAM	Italy	265	271	127	187
National Centre for Scientific Research Demokritos	DEMOKRITOS	Greece	85	132	9	23
Dana-Farber Cancer Institute	DFCI	USA	82	65	81	46
German Cancer Research Center	DKFZ	Germany	19	36	10	14
Epidemiological Study of Familial Breast Cancer	EMBRACE	UK/Ireland	908	795	867	770
Fox Chase Cancer Center	FCCC	USA	49	26	31	11
Fundación Pública Galega de Medicina Xenómica	FPGMX	Spain	41	67	31	44
Ghent University Hospital	G-FAST	Belgium	69	121	87	76
German Familial Breast Group	GC-HBOC	Germany	675	1,168	407	646
Genetic Modifiers of cancer risk in BRCA1/2 mutation carriers	GEMO	France/USA	630	842	328	590

Georgetown University	GEORGETOWN	USA	6	5	0	0
Hospital Clinico San Carlos	HCSC	Spain	85	56	77	76
Helsinki Breast Cancer Study	HEBCS	Finland	67	53	63	67
Genen Omgeving studie van de werkgroep Hereditiair Borstkanker Onderzoek Nederland	HEBON	Netherlands	491	374	401	199
Molecular Genetic Studies of Breast- and Ovarian Cancer in Hungary	HUNBOCS	Hungary	101	179	48	65
University Hospital Vall d'Hebron	HVH	Spain	56	62	65	63
Institut Català d'Oncologia	ICO	Spain	150	130	163	185
International Hereditary Cancer Centre	IHCC	Poland	121	77	0	0
Iceland Landspitali – University Hospital	ILUH	Iceland	0	0	29	111
INterdisciplinary HEalth Research Internal Team BReast CAncer susceptibility	INHERIT	Canada (Quebec)	52	37	46	34
Istituto Oncologico Veneto	IOVHBOCS	Italy	93	111	53	113
Portuguese Oncology Institute-Porto Breast Cancer Study	IPOBCS	Portugal	79	36	73	88
Kathleen Cuningham Consortium for Research into Familial Breast Cancer	KCONFAB	Australia	356	368	273	295
University of Kansas Medical Center	KUMC	USA	3	24	0	12
Mayo Clinic	MAYO	USA	127	122	54	74
McGill University	MCGILL	Canada (QUEBEC)	30	24	20	14
Memorial Sloane Kettering Cancer Center	MSKCC	USA	195	185	189	167
General Hospital Vienna	MUV	Austria	266	268	120	142
National Cancer Institute	NCI	USA	109	42	62	21
N,N, Petrov Institute of Oncology	NNPIO	Russia	22	44	0	2
NorthShore University HealthSystem	NORTHSHORE	USA	40	40	36	19
NRG Oncology	NRG_ONCOLOGY	USA/Australia	153	166	147	145
Ontario Cancer Genetics Network	OCGN	Canada	133	71	107	64
The Ohio State University Comprehensive Cancer Center	OSU CCG	USA	34	50	43	56
Odense University Hospital	OUH	Denmark	358	192	258	167
Università di Pisa	PBCS	Italy	39	49	1	6
Sheba Medical Centre	SMC	Israel	99	66	47	33
Swedish Breast Cancer Study	SWE-BRCA	Sweden	237	190	39	25
University of Chicago	UCHICAGO	USA	51	43	28	29
University of California San Francisco	UCSF	USA	60	33	35	28
UK and Gilda Radner Familial Ovarian Cancer Registries	UKGRFOCR	UK	40	13	12	4
University of Pennsylvania	UPENN	USA	220	240	178	168
Cancer Family Registry University of Pittsburg	UPITT	USA	77	77	56	43
University of Texas MD Anderson Cancer Center	UTMDACC	USA	18	25	28	40
Victorian Familial Cancer Trials Group	VFCTG	Australia	104	103	131	70
Women's Cancer Program at Cedars-Sinai Medical Center	WCP	USA	137	51	51	18
**Total**			**7,790**	**7,889**	**5,375**	**5,608**

Supplementary Figure 1. Un-cropped blots of Figure 1.

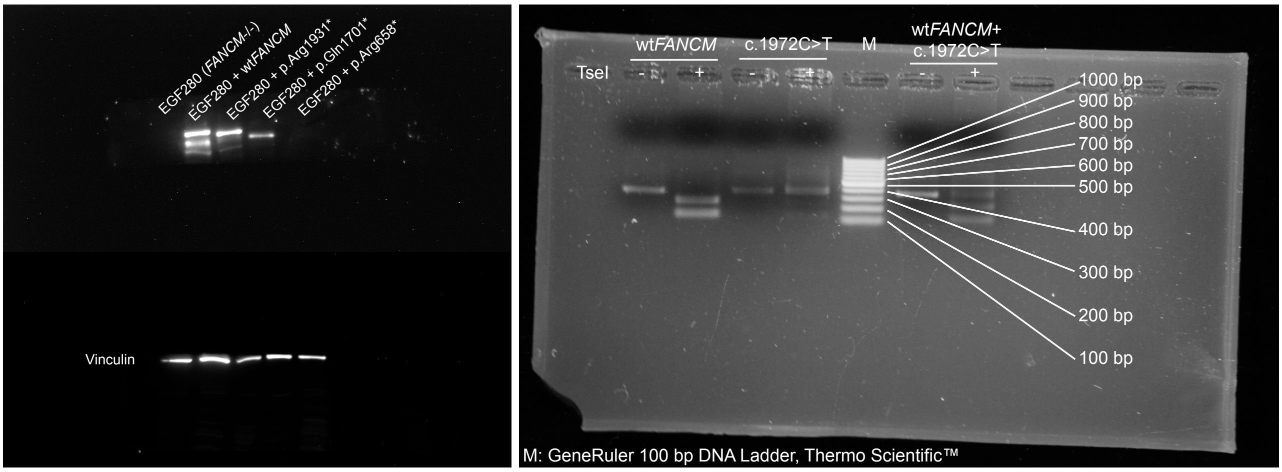

Supplementary Figure 2. Cluster plots of *FANCM*:p.Arg658*, p.Gln1701*, and p.Arg1931* truncating variants from the BCAC (**a**) and CIMBA (**b**) genotyping data. In two BCAC studies from Sweden, a case who developed triple negative breast cancer at age 66, and a control ascertained at age 34 both homozygous for the p.Gln1701* were found.

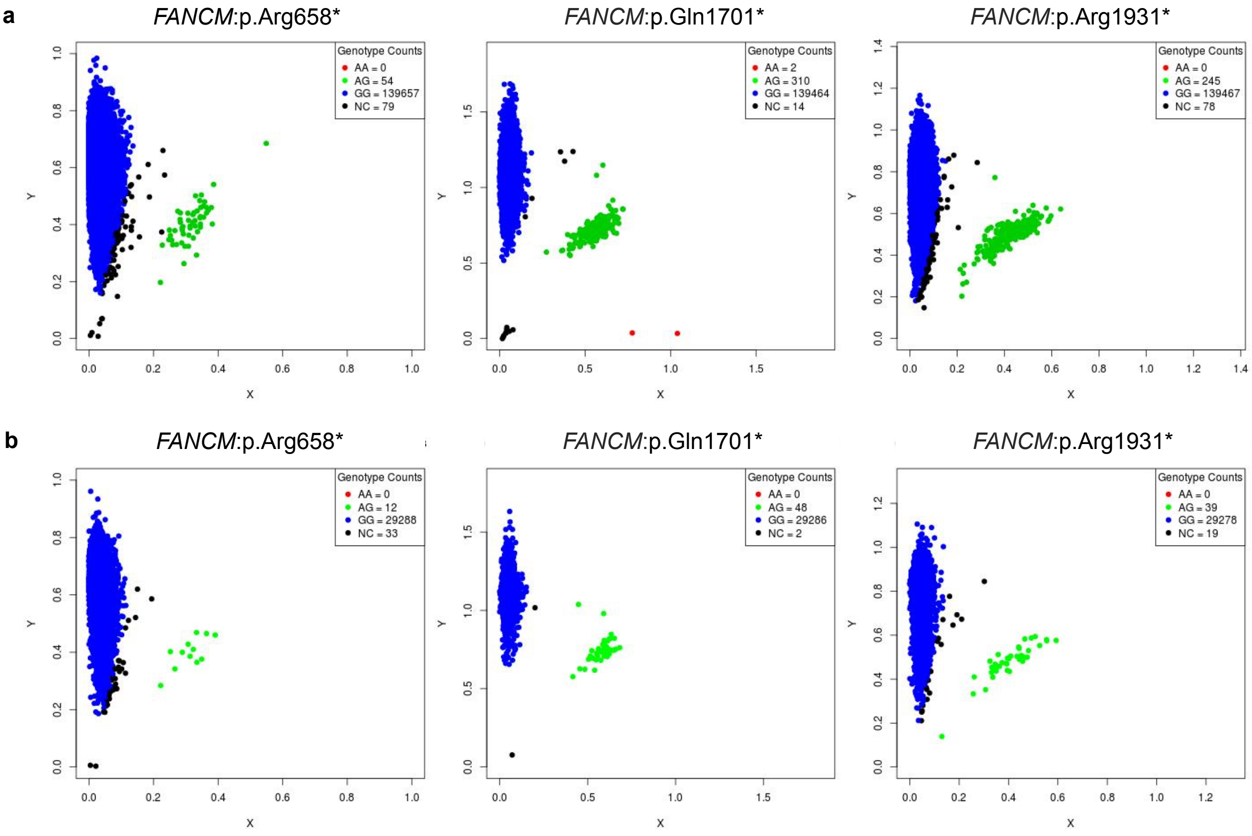

Supplement: Supplementary file 1 — Supplementary material [file 41523_2019_127_MOESM1_ESM.pdf]
